# Time trend of global uterine cancer burden: an age-period-cohort analysis from 1990 to 2019 and predictions in a 25-year period

**DOI:** 10.1186/s12905-023-02535-5

**Published:** 2023-07-21

**Authors:** Liu Yang, Yue Yuan, Rongyan Zhu, Xuehong Zhang

**Affiliations:** 1 The First Clinical Medical College, Lanzhou University, Lanzhou, 730000 China; 2 Department of Center for Reproductive Medicine, The First Hospital of Lanzhou University, No. 11, Donggang Road (West), Cheng-Guan District, Lanzhou, 730000 China

**Keywords:** Uterine cancer, Global burden of Disease, Incidence, Death, Age-period-cohort model, Forecasting

## Abstract

**Background:**

Uterine cancer remains a serious medical problem worldwide. This study aimed to explore the global time trends of uterine cancer burden using the age-period-cohort model and forecast incidence to 2044.

**Methods:**

Data were downloaded from the Global Burden of Disease 2019. The age-period-cohort model was used to estimate age, period and birth cohort effects. We also predict uterine cancer incidence to 2044.

**Results:**

Globally, there were 435,041 incident cases (95% UI: 245,710 to 272,470) and 91,640 deaths of uterine cancer (95% UI: 39,910 to 44,140) in 2019. During the past 30 years, the age-standardized incidence and death rates increased by 15.3% and decreased by 21.6%, respectively. Between 1990 and 2019, the high-sociodemographic index region had the highest overall annual percentage changes. The age effect showed the uterine cancer incidence rate first increased and then decreased with age. The period and cohort relative rate ratio showed upward trends during the study period. Incident cases of uterine cancer may increase to more than six hundred thousand in 2044.

**Conclusion:**

Uterine cancer causes a high disease burden in high-income regions and the global incidence may continue to increase in the future. Improving awareness of risk factors and reducing the proportion of the obese population are necessary to reduce future burden.

**Supplementary Information:**

The online version contains supplementary material available at 10.1186/s12905-023-02535-5.

## Introduction

Uterine cancer is the most common tumor in female reproductive organs, mainly occurring in postmenopausal women [1]. There were 417,000 new diagnoses globally in 2020, with cases doubling in women under 40 years of age [2]. Uterine cancer-related mortality has increased by an average of 1.9% per year from 1971 to 2014 [3]. It is expected to rank fourth in new cancer cases and sixth in deaths among females in the USA in 2023 [4]. Over the past few decades, the overall incidence of uterine cancer has increased by 132%, and it poses a serious medical problem worldwide [2]. As the world’s population grows, the population ages and the prevalence of risk factors increases, and the disease burden of uterine cancer may continue to increase.

Some studies have described the epidemiological features of uterine cancer at the regional or national level [5–9]. Cancer is an age-related disease; in addition, the epidemiology of the disease may be influenced by the time period and the birth cohort time of the population. However, studies focusing on the effects of age, period, and cohort on uterine cancer incidence are still lacking. Additionally, few studies have focused on predicting the future incidence trends of uterine cancer. Thus, using age-period-cohort analysis to assess the independent effects of age, period, and cohort on disease incidence and mortality, and predicting future epidemiological trends of uterine cancer may be helpful for cancer prevention and control.

The Global Burden of Disease Study 2019 (GBD 2019) assessed 369 diseases and injuries worldwide, providing data to analyze the epidemiological patterns and features of uterine cancer [10]. In this study, we conducted a systematic analysis to describe the time trends and patterns of uterine cancer based on an age-period-cohort (APC) model and present forecasts for global trends up to 2044, aiming to provide new viewpoints on this gynecological cancer.

## Methods

### Data source

Epidemiological data of uterine cancer were downloaded from GBD 2019: http://ghdx.healthdata.org/gbd-results-tool (access on December 1, 2022), including annual count and age-standardized rate (ASR) of incidence, death and disability adjusted of life year (DALY) from 1990 to 2019. Cause-specific deaths attributed to uterine cancer were referred to the following International Classification of Diseases and Injuries (ICD) codes: C54-C54.3, C54.8-C54.9, Z85.42, Z86.001 (ICD-10) and 182-182.9 (ICD-9). More information about the data source, inputs and estimation models are available in the previous publications [10, 11]. This study followed the “Guidelines for Accurate and Transparent Health Estimates Reporting” reporting guideline for cross-sectional studies [12]. For GBD studies, a waiver of informed consent was reviewed and approved by the Institutional Review Board of the University of Washington [13]. The information about ethical standards is available on the GBD official website (http://www.healthdata.org/gbd/2019).

### Sociodemographic index (SDI)

The SDI is a comprehensive indicator based on the overall fertility rate, educational attainment, and lagging per capita income distribution in a region or country, which ranges from 0 to 1 [14]. The closer the SDI value is to 1, the more developed the social economy of the region/country is. All countries and territories were classified into five categories according to SDI values. The SDI values of all regions, countries and territories can be downloaded at: https://ghdx.healthdata.org/record/ihme-data/gbd-2019-socio-demographic-index-sdi-1950-2019.

### APC analysis

The APC model was used to evaluate the impact of age, period, and birth cohort effects on health outcomes in epidemiological studies [15]. The age effect explains the difference in the incidence of uterine cancer in different age groups caused by age-related factors. Periodic effects refer to the influence of various factors during the study period (1990 to 2019) on uterine cancer incidence, such as social progress and development of medical levels. Cohort effects are changes in cancer incidence due to exposure to different risk factors in a population of different birth years. We used the Age-Period-Cohort Analysis Tool (https://analysistools.cancer.gov/apc) to calculate relative risk their and 95% confidence intervals (CIs) to evaluate age, period, and birth cohort effects on cancer incidence [16].

In a typical age-period-cohort model, the age and period intervals must all be equal. Due to the age group of five-year intervals in GBD 2019, we arranged the incidence and population data into successive five-year periods (1990 to 1994, 1995 to 1999, 2000 to 2004, 2005 to 2009, 2010 to 2014, and 2015 to 2019), with 1990 to 1994 as the reference period. We also used age groups with five-year age intervals from GBD 2019 (20 to 24, 25 to 29, 30 to 34, etc.), with 20 to 24 years as the reference age group. The assessment indicators in the APC model from the web tool include age-specific rates, period rate ratios (RRs), cohort rate RR, net drift and local drifts [16].

### Data analysis

All data analysis was performed in R software (version 4.2.2). To clarify the impact of population growth, age structure and other factors on disease burden, we analyzed the DALY change from 1990 to 2019 by decomposition analysis [17]. The RRs and their 95% CIs were used to assess the effect of period and cohort on cancer incidence. The Wald chi-squared test in the APC model was used to test the significance of the estimated parameters. We used the “Nordpred” package in R software to project the future trend of uterine cancer incidence [18]. “Nordpred” is a well-established estimation method for cancer incidence and mortality prediction, and has been validated and used in many publications [18–20]. All rates in this study are reported per 100,000 population. *P* value < 0.05 was considered statistically significant.

## Results

### Overview of uterine cancer burden

Globally, there were 435,041 new incident cases (95% UI: 245,710 to 272,470) and 91,640 deaths from uterine cancer (95% UI: 39,910 to 44,140) in 2019. Uterine cancer was responsible for 2,329,074 DALYs (95% UI: 2,092,947 to 2,560,886) in 2019. From 1990 to 2019, incident cases and deaths increased by 132% and 63%, respectively. The age-standardized incidence rate (ASIR) showed an upward trend (percent change: 15.3%, 95% CI: 5.9–26%), while the age-standardized death rate (ASDR) showed a downward trend (percent change: -21.6%, 95% CI: -26.8% to -14.7%) (Table 1, Figure S1). Uterine cancer incidence and death also varied by age group worldwide in 2019 (Figure S2). With increasing age, the ASIR showed a trend of first increasing and then decreasing. The global ASIR of uterine cancer peaked at the age of 65 to 69 years. For ASDRs, it always increased with age.

**Table 1:** Age-standardized incidence rate and its change trends of uterine cancer, 1990 to 2019

	ASIR in 1990	ASIR in 2019	Percentage change of rate, (%)
Global	8.67 (8.1–9.08)	9.99 (9.12–11.02)	15.3 (5.9 to 26)
Different SDI			
High SDI	13.82 (13.36–14.15)	19.16 (16.94–21.48)	38.7 (23.1 to 55.7)
High-middle SDI	11.66 (11.15–12.19)	13.87 (12.4-15.37)	18.9 (7.3 to 31.6)
Middle SDI	4.45 (3.48–5.05)	5.7 (4.72–6.67)	28.2 (8.8 to 57.7)
Low-middle SDI	3.09 (2.53–3.71)	3.94 (3.36–4.8)	27.5 (9.9 to 50.3)
Low SDI	2.78 (2.2–3.5)	3.43 (2.81–4.21)	23.4 (4 to 53)
GBD Region			
Andean Latin America	7.57 (6.06–8.74)	9.75 (7.65–12.67)	28.8 (-1.2 to 69.2)
Australasia	9.46 (8.88–10.03)	11.26 (9.13–13.94)	19 (-3.5 to 48.3)
Caribbean	11.61 (10.82–12.43)	17.83 (15.11–20.97)	53.5 (29.3 to 79.8)
Central Asia	10.77 (10.18–11.44)	11.72 (10.47–13.11)	8.8 (-3.7 to 22.7)
Central Europe	13.83 (13.32–14.55)	20.52 (17.68–23.86)	48.4 (28.3 to 72.5)
Central Latin America	4.1 (3.94–4.26)	6.4 (5.39–7.58)	56 (31 to 85.4)
Central Sub-Saharan Africa	2.82 (2.11–3.96)	3.01 (2.14–4.32)	6.6 (-24.5 to 46.6)
East Asia	5.17 (3.92–6.33)	6.55 (5.07–8.8)	26.8 (-7.3 to 86)
Eastern Europe	20.42 (19.56–21.44)	27.5 (23.25–32.58)	34.7 (13.9 to 60.1)
Eastern Sub-Saharan Africa	3.27 (2.32–4.01)	3.7 (2.64–4.57)	13 (-7.3 to 39.3)
High-income Asia Pacific	6.74 (5.95–7.08)	11.32 (9.21–13.68)	67.9 (36 to 108.3)
High-income North America	19.15 (18.49–19.68)	27.82 (23.11–33.44)	45.3 (21.1 to 74.7)
North Africa and Middle East	3.1 (2.36–3.73)	5.41 (3.71–6.39)	74.7 (37 to 132.3)
Oceania	6.75 (4.79–8.38)	8.58 (4.86–11.31)	27.1 (-8.1 to 65.6)
South Asia	2.17 (1.71–2.74)	2.94 (2.34–3.61)	35.6 (9.6 to 73.2)
Southeast Asia	4.7 (3.36–5.46)	6.23 (4.1–7.47)	32.5 (9 to 58.8)
Southern Latin America	6.97 (6.59–7.36)	8.23 (6.43–10.38)	18.1 (-8.9 to 50.1)
Southern Sub-Saharan Africa	3.48 (2.82–4.05)	5.08 (3.57–5.87)	45.9 (20.8 to 71.3)
Tropical Latin America	5.92 (5.62–6.2)	6.97 (6.5–7.48)	17.7 (10 to 26.6)
Western Europe	13.14 (12.68–13.52)	19.62 (16.98–22.47)	49.3 (29.5 to 71.3)
Western Sub-Saharan Africa	2.13 (1.73–3.16)	2.64 (2.13–3.58)	24.1 (1.4 to 51.7)

ASIR: age-standardized incidence rate; SDI: socio-demographic index

In 2019, the highest ASIR and ASDR were found in the American continent: high-income North America (ASIR:27.82) and Caribbean (ASDR: 5.67), respectively. All 21 GBD regions showed upward trends of ASIR, with North Africa and Middle East increasing fastest during the past 30 years (percent change = 74.7%, 95% CI: 37.0–132.3%). Except for Oceania, Caribbean, Southern Sub-Saharan Africa, high-income North America and Western Sub-Saharan Africa, other GBD regions showed downward trends of ASDR, and the ASDR declined fastest in East Asia (percent change = -49.3%, 95% CI: -61.7% to -27.2%) (Table 1, Table S1).

**Fig. 1:**
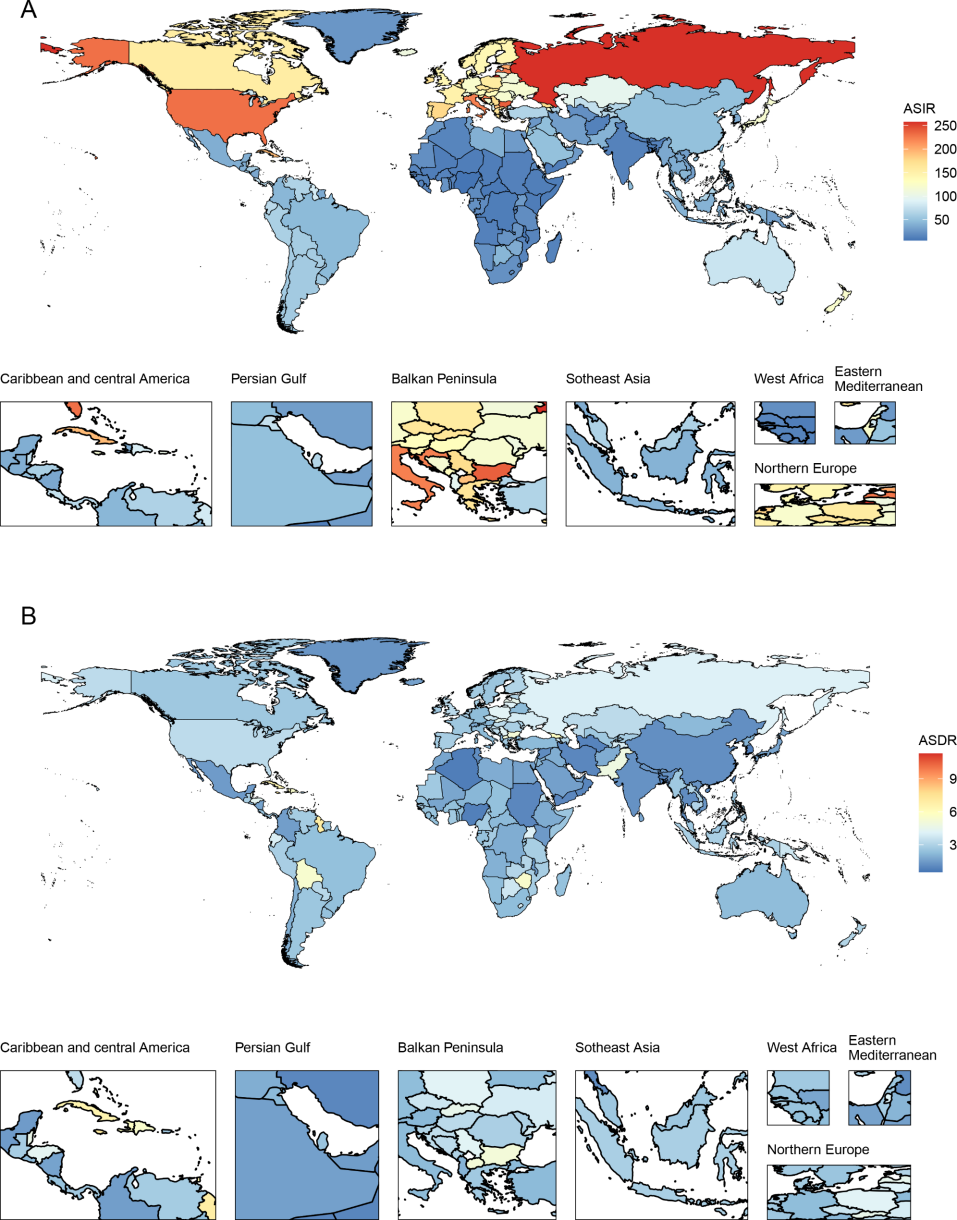
The age-standardized incidence and death rate of uterine cancer among all countries/territories in 2019. ASIR: age-standardized incidence rate; ASDR: age-standardized death rate

At the national level, the United States of America (incidence: 80,070, deaths: 10,260) and China (incidence: 66,744, deaths: 12,222) had the highest incident cases and deaths, respectively. The ASIR and ASDR also varied among different countries/territories (Fig. 1). In 2019, the Northern Mariana Islands had the highest ASIR, followed by the Russian Federation and Bulgaria. Grenada, American Samoa and Saint Vincent and the Grenadines were the top three countries/territories that had the highest ASDRs in 2019. Counts and ASRs of uterine cancer incidence and mortality in 1990 and 2019 are shown in Tables S2 and S3.

### Burden of uterine cancer among SDI

**Fig. 2:**
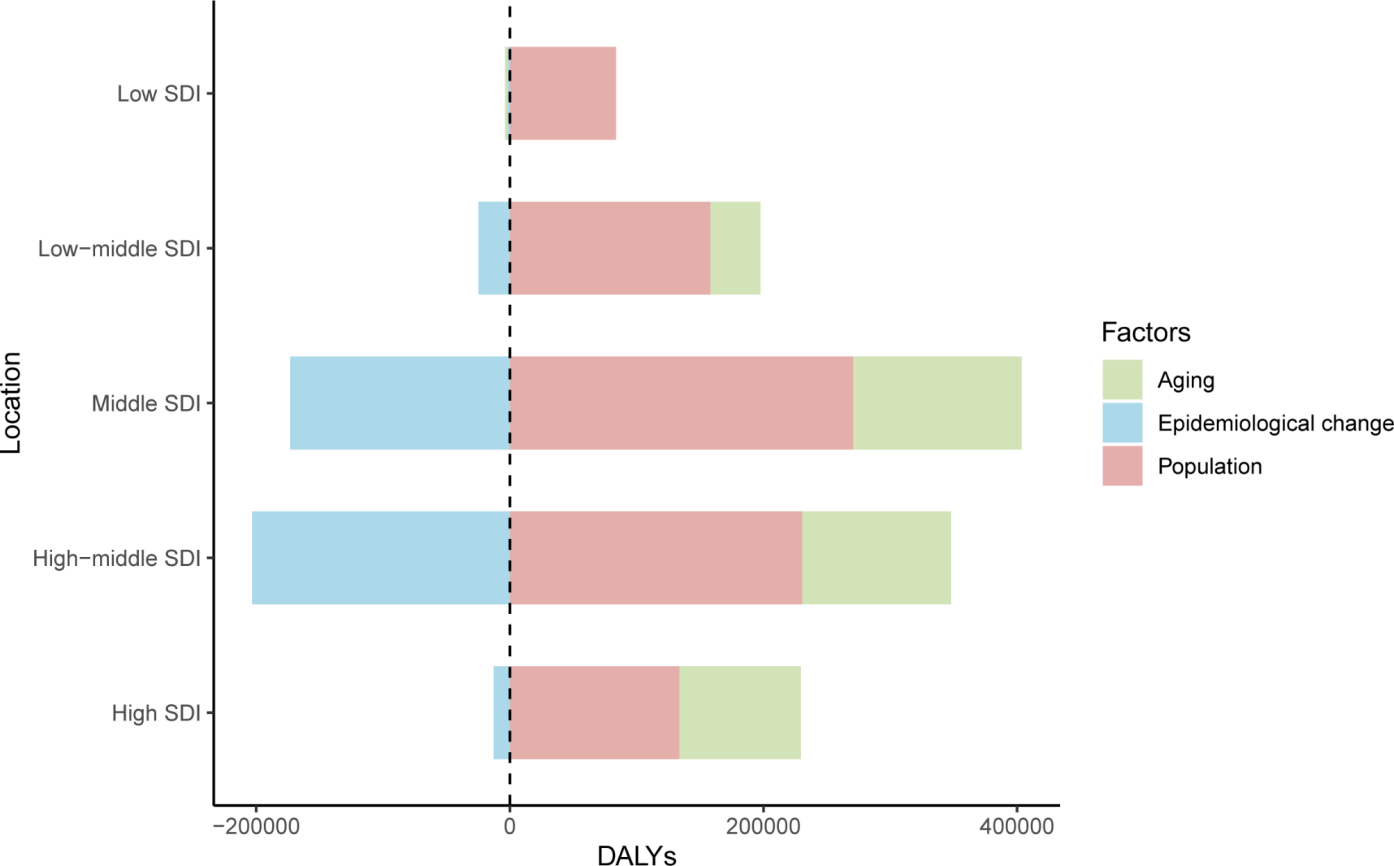
Decomposition analysis of uterine cancer DALYs between 1990 to 2019, by SDI. DALYs: disability adjusted of life year; SDI: sociodemographic index

In 2019, among the five SDI quintiles, the ASIR of uterine cancer decreased from the high SDI quintile (19.16) to the low SDI quintile (3.43). The highest ASDR was observed in high SDI region (2.52) and the lowest in the middle SDI region (1.61). The ASRs in 16 age groups among different SDI regions are shown in Figure S2. Figure 2 demonstrates the decomposition analysis of age-related DALYs between 1990 and 2019 by SDI. Except in the low SDI region, aging has contributed to the increase in DALYs in the past 30 years, and the high-middle SDI region is most affected by an aging population (81.18%). The proportion of population growth was highest in the high-middle SDI region (159.24%), followed by the middle SDI (117.44%) and low SDI (104.76%) regions (Fig. 2). The relationships between ASRs and SDI among different regions are shown in Figure S3.

### Time trends in uterine cancer incidence across different age groups

**Fig. 3:**
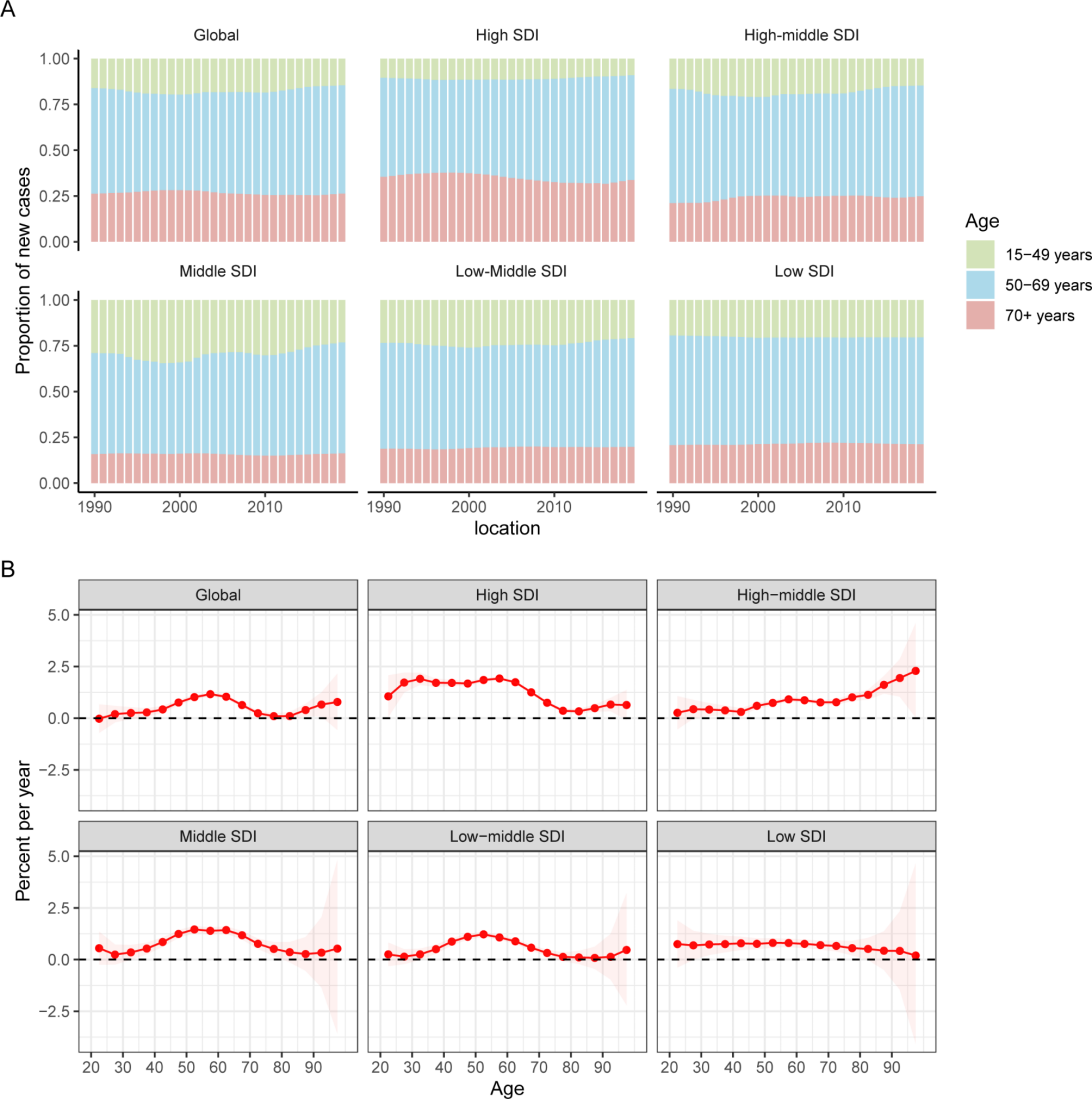
Age distribution of incident cases and local drifts of uterine cancer incidence by SDI quintiles, 1990 to 2019. SDI: sociodemographic index

Temporal changes in the age distribution of incidence are presented in Fig. 3A. Globally, people aged 50 to 69 years accounted for the majority of incident cases of uterine cancer from 1990 to 2019. Young and middle-aged people (< 50 years) in the middle-SDI quintile had the highest proportions of incident cases among the five SDI quintiles, which were nearly 25%.

Figure 3B shows the annual percentage change in the uterine cancer incidence for each age group. Globally, uterine cancer incidence showed increasing trends. The most significant increase occurred in the 55 to 60 years group (local drift = 1.63%, 95% CI: 1.07–1.26%). The percentage change increased with age in the high-middle SDI population and vice versa in the low SDI region. In the young and middle-aged population (< 50 years), the high SDI region had the fastest increase in the uterine cancer incidence rate. For elderly individuals, the high-middle SDI region had the highest local drift.

### APC analysis

**Fig. 4:**
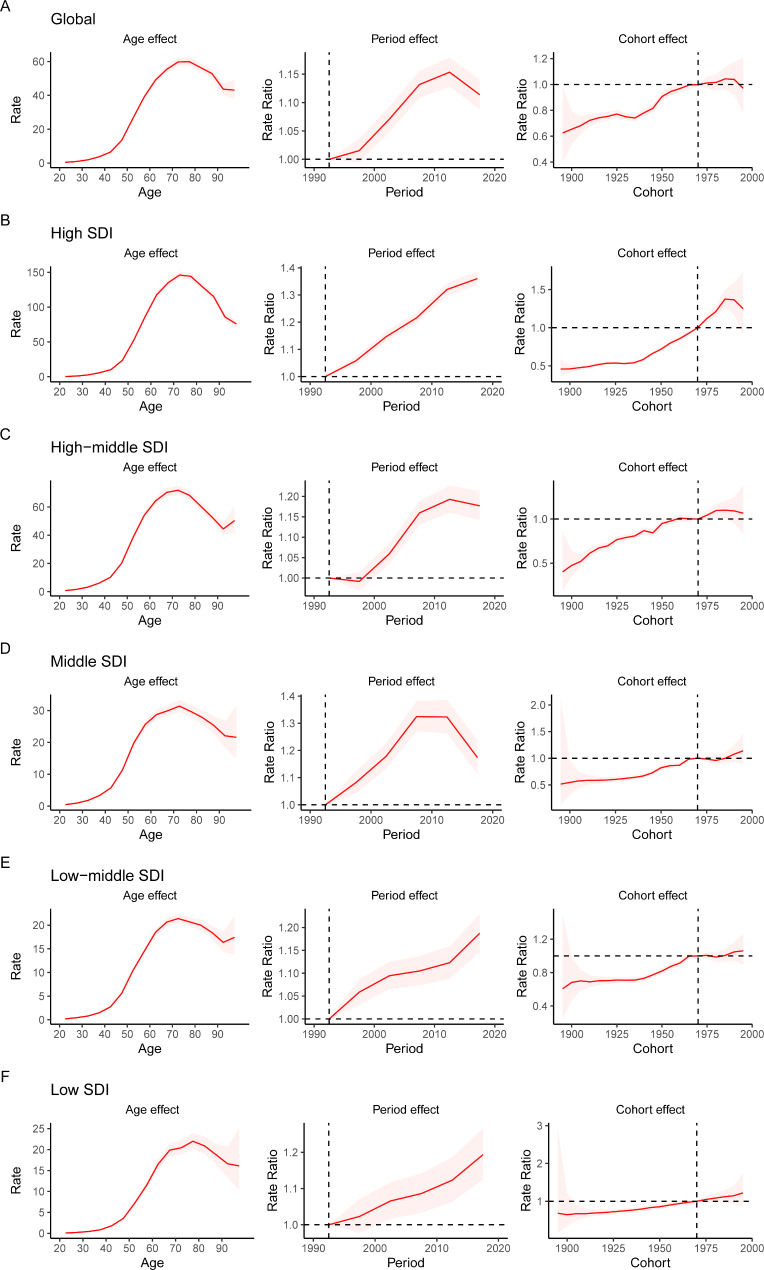
Age, period and cohort effects on uterine cancer incidence by SDI quintiles. SDI: sociodemographic index

The APC mode by SDI quintile is shown in Fig. 4. We found similar patterns in age effects across five SDI quintiles, with the incidence first increasing and then decreasing with age. Compared to other regions, high-SDI region showed an overall higher incidence rate across all age groups. Period effects showed that compared to the reference period (1990 to 1994), the RRs of incidence presented upward trends from 1990 to 2019 in five SDI quintiles. The high SDI region had the highest period RR in the latest period (2015 to 2019, RR = 1.36, 95% CI: 1.34 to 1.39). Globally, there was an overall increasing risk from early birth cohorts to the latest birth cohorts. Similar to period effects, increasing cohort effects were more obvious in high SDI region. Compared with people born in the referent 1970 cohort, the cohort RR for people born in the 1995 cohort ranged from 1.26 (95% CI: 0.91 to 1.75) in high SDI region to 1.06 (95% CI 0.89 to 1.27) in low-middle SDI region.

### Prediction to 2044

**Fig. 5:**
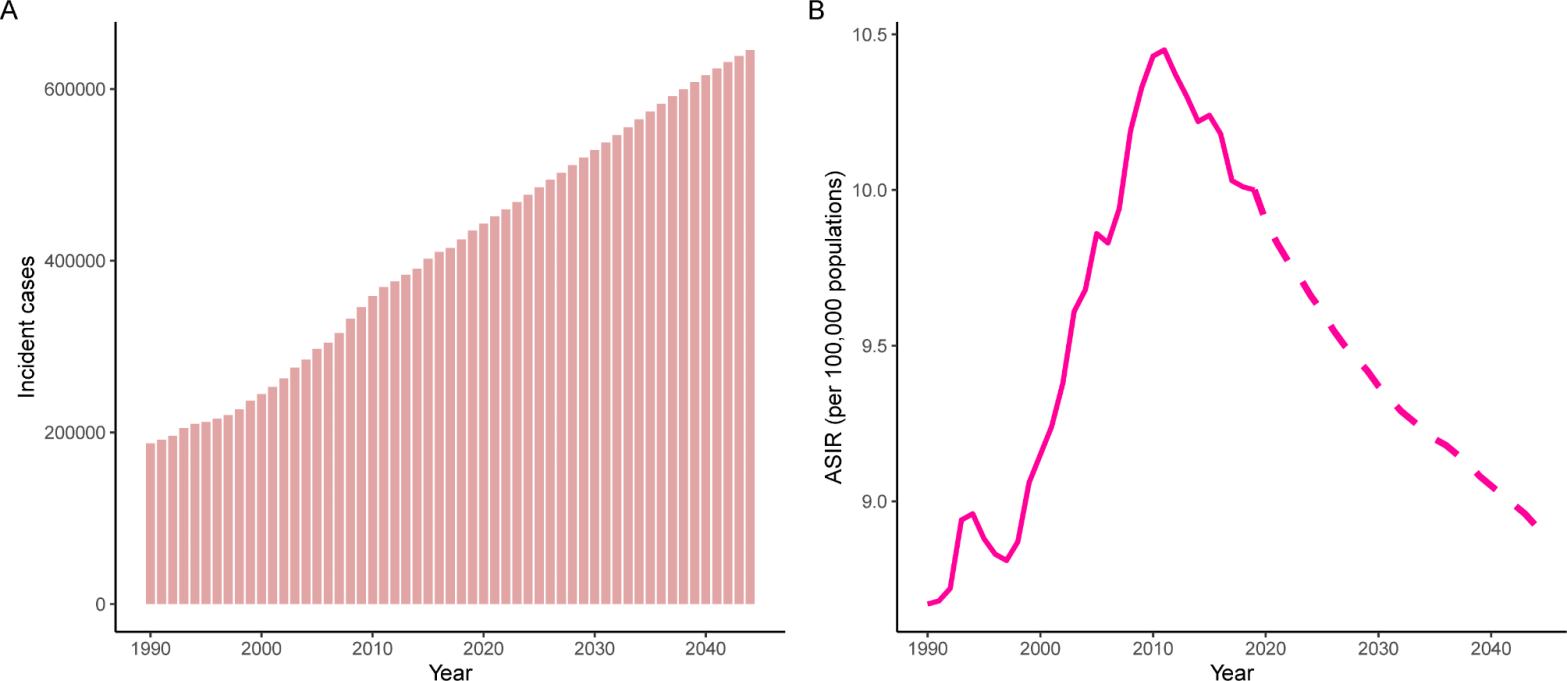
Trends in **(A)** number and **(B)** age-standardized rate of incidence for uterine cancer worldwide from 1990 to 2044. Observed rates are plotted with solid lines and predicted rates are plotted with dashed lines

**Fig. 6:**
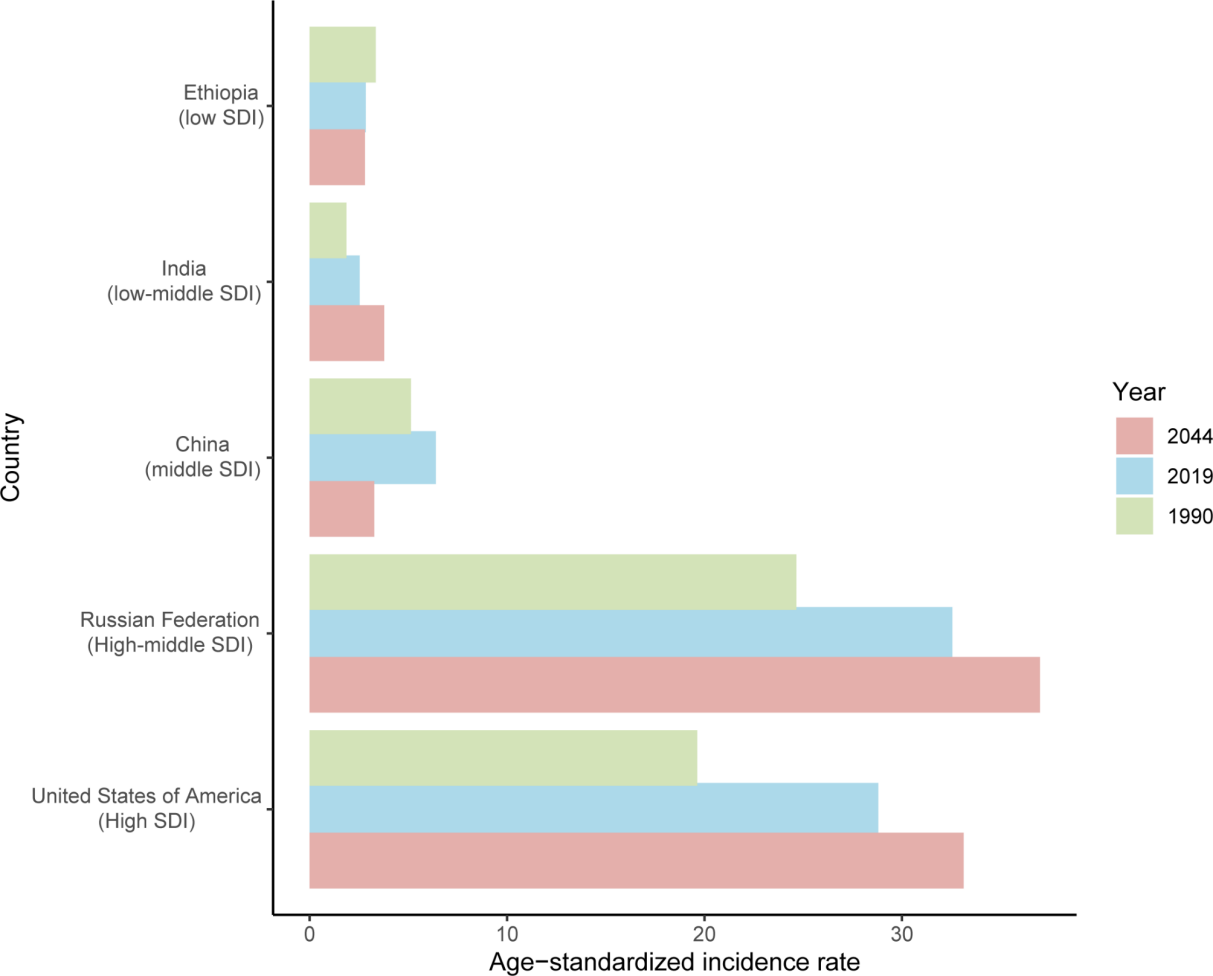
Age-standardized incidence rates of uterine cancer in 1990 and 2019, and predicted to 2044 in five countries. SDI: sociodemographic index

The incident cases and incidence rate of uterine cancer are predicted to continue to increase in the next 25 years. As shown in Fig. 5A, the number of incident cases of uterine cancer may increase to more than six hundred thousand in 2044, which will be 1.48 times that in 2019. The ASIR will show a downward trend, from 10.0 to 2019 to 8.92 in 2044 (Fig. 5B). We also predict future ASIRs of uterine cancer in several exemplary countries across SDI quintiles (Fig. 6). The results showed that the ASIR may still be significantly higher in high- and high-middle-SDI countries (the USA and Russia). Developing countries with relatively low ASIRs will show declining trends (China and Ethiopia). The ASIR in 2044 in India will be nearly two times that in 1990.

## Discussion

Uterine cancer shows an increasing incidence and disease-associated mortality worldwide. Socioeconomic and geographical differences are important determinants of uterine cancer incidence and mortality [21]. The results from our study demonstrated that the incident cases and ASIRs of uterine cancer showed an increasing trend, and the higher the SDI, the higher the incidence; according to our forecasting, ASIRs of uterine cancer will continue to increase in the next 25 years. This highlights the urgency of the establishment of updated cancer prevention strategies across regions and countries.

The risk of uterine cancer increases with age and body mass index (BMI) [2]. From our decomposition analysis, we found that population aging and population growth have contributed to the increase in DALYs from 1990 to 2019. Despite decreasing epidemiological changes among the five SDI quintiles, DALYs were still high. Of the various common cancers, uterine cancer has the strongest relationship with overweight and obesity [2]. Each 5-unit increase in BMI was associated with a 50% increased risk of endometrial cancer [22]. In addition, among patients with endometrial cancer, patients with high BMI had a higher disease-specific mortality rate [23]. Estrogen excess or progesterone deficiency is one of the main causes of uterine cancer, and one of the major causes of the estrogen/progesterone imbalance is obesity. Moreover, hyperinsulinemia is another mechanism that causes endometrial cancer: the binding of insulin to insulin-receptors can stimulate the growth of endometrial stromal cells [24].

Our study shows that the incidence of uterine cancer is higher in high-income regions or countries, especially in North America and Europe. The risk factors for uterine cancer showed a distribution pattern that matched socioeconomic development. First, people in economically developed areas tend to have a highly processed and high-calorie diet, such as red meat, fat and sugary foods, which is a major cause of overweight and obesity, leading to an increased risk of uterine cancer. The study showed that the risk of endometrial cancer is increased in people with a high glycemic load diet [25]. Next, people in countries with faster economic development seem to have less opportunity and time for physical exercise, leading to overweight and obesity.

We performed the APC analysis and quantified the annual percentage change on uterine cancer incidence. The age effect increased from the youngest age group to the 70 to 74 age group and subsequently decreased. Cancer seems to be a disease of the elderly because there is a link between cancer and cellular aging [26]. Aging can lead to changes in sex hormone levels in women. Moreover, the prevalence of obesity and diabetes is higher among older people [27, 28].

The period effect on uterine cancer incidence markedly increased globally, especially in high SDI regions, which may be explained by external factors, such as socioeconomic level, medical technology and lifestyle. During the past 30 years, social and economic development has been rapid in most regions and countries around the world. There has been a significant increase in the consumption of energy/fat dense foods. These dietary factors can increase body fat accumulation and hence the risk of uterine cancer development and progression [24]. Globally, the proportion of adults with a BMI of 25 kg/m^2^ or greater increased by nearly 10% in females from 1980 to 2013 [27]. The risk and burden of uterine cancer also seem to vary with BMI change globally. Women’s reproductive characteristics, such as advanced maternal age and cesarean section, and reproductive factors that increase lifetime exposure to unopposed estrogen (such as nulliparity) are also risk factors for endometrial neoplasia [2, 29]. Additionally, the improvement of screening technology will also increase the reported incidence rate of uterine cancer. In countries with high medical levels, a comprehensive uterine cancer diagnosis system that contained imaging, tumor markers, hysteroscopy and gene detection improved the detection rate of disease [21]. In contrast, low-income regions or countries have poor health care and inadequate disease-registration systems, which may lead to low incidence in registration.

The cohort effect demonstrated the change in the incidence of uterine cancer caused by the different types and levels of exposure of people at different ages of birth. Since the reference birth cohort (1970 to 1974), the cohort RRs first increased and then decreased. Similar to the period effects, the increased risk was associated with bad dietary habits. However, a standardized disease prevention and medical care system has been established in high-income countries, and these people have been paying increasing attention to cancer prevention in recent years. The later the cohort was born, the better the health education people can be accepted, so health consciousness has improved in young people, and they may pay more attention to physical examination and chronic disease prevention. A more scientific lifestyle reduces the exposure of risk factors for uterine cancer.

We also predict the future incidence pattern of uterine cancer at the global level. As the population grows and ages in the coming decades, the number of incident cases of uterine cancer will continue to increase. Cancer prevention and early cancer screening are currently the priority tasks of cancer-related public health and medical policies. With the popularization of science education and the promotion of a healthy lifestyle, there may be much more understanding of uterine cancer for people worldwide in the future. Public health interventions that decrease the prevalence of overweight and obesity may have a positive impact on decreasing incidence rates of uterine cancer. Studies have shown that the successful treatment of obesity can reduce endometrial cancer risk [30, 31]. Risk prediction scores or models that combine genetic factors, clinical features, and reproductive factors and will provide new insight into uterine cancer screening and prevention interventions in the future. Comprehensive treatment strategies, including surgery, chemotherapy, immunotherapy and combination therapy for uterine cancer should also be further refined in the future to reduce mortality.

In the future, we should focus on achieving and maintaining a healthy body weight to reduce the risk of uterine cancer. Given the large variations in disease burden by SDI, future strategies to prevent and reduce the uterine cancer burden should be developed based on country-specific social development status. In some high-income regions or countries, people should adopt healthier eating patterns and strengthen physical exercise to reduce risk factors for uterine cancer, such as obesity. Although low-SDI countries do not have a high disease burden of uterine cancer, more improved early-stage cancer screening programs, accurate cancer diagnosis tools and health education for women are also needed.

There are some limitations in our study. First, the GBD Study tends to underestimate some data in low-income regions or countries due to a lack of advanced and accurate diagnostic techniques. Moreover, data in GBD 2019 were estimated by the DisMod-MR 2.1 model, and there might be some derivations and uncertainty values. Next, due to the lack of individual data, epidemiological data of uterine cancer classified by histological stage were not available in this study. Future work should focus on high-risk populations and high-burden regions or countries. Greater efforts and improvements are still needed to improve disease data registration and collection in developing countries. The economic burden of uterine cancer should also be further explored and collected.

## Conclusion

Uterine cancer poses a serious health problem worldwide and incident cases may continue to increase in the next 25 years. More measures and efforts must be put into cancer prevention and treatment strategies for uterine cancer, including reducing the obesity population, early cancer screening, and next generation of cancer therapies.

## Electronic supplementary material

Below is the link to the electronic supplementary material.

Additional File 1
Figure S1 Counts and age-standardized rates of uterine cancer incidence and death at the global level, 1990 to 2019.
Figure S2 Age patterns of incidence and deaths of uterine cancer by SDI in 2019.
Figure S3 Age-standardized rates of uterine cancer globally and for 21 regions by SDI, 1990 to 2019.
Table S1 Age-standardized death rate and its change trends of uterine cancer, 1990 to 2019.
Table S2 The incidence information of uterine cancer in 1990 and 2019 among all countries/territories.
Table S3 The death information of uterine cancer in 1990 and 2019 among all countries/territories.

## Data Availability

The datasets generated and/or analysed during the current study are available in the Global Health Data Exchange query tool, which is a publicly available source (https://vizhub.healthdata.org/gbd-results/).

## Abbreviations

APC: Age-period-cohort
ASIR: Age-standardized incidence rate
ASDR: Age-standardized death rate
ASR: Age-standardized rate
BMI: Body mass index
CI: Confidence interval
DALY: Disability adjusted of life year
ICD: International Classification of Diseases and Injuries
GBD: Global Burden of Disease
RR: Rate ratio
SDI: Sociodemographic index
