# Supplementary material for: Time trend of global uterine cancer burden: an age-period-cohort analysis from 1990 to 2019 and predictions in a 25-year period
Source: BMC Womens Health. 2023 Jul 21;23:384. doi: 10.1186/s12905-023-02535-5 (PMC10362563; doi:10.1186/s12905-023-02535-5)
Supplement: Supplementary file 1 — Additional File 1 Figure S1 Counts and age-standardized rates of uterine cancer incidence and death at the global level, 1990 to 2019. Figure S2 Age patterns of incidence and deaths of uterine cancer by SDI in 2019. Figure S3 Age-standardized rates of uterine cancer globally and for 21 regions by SDI, 1990 to 2019. Table S1 Age-standardized death rate and its change trends of uterine cancer, 1990 to 2019. Table S2 The incidence information of uterine cancer in 1990 and 2019 among all countries/territories. Table S3 The death information of uterine cancer in 1990 and 2019 among all countries/territories. [file 12905_2023_2535_MOESM1_ESM.pdf]

Supplementary Data

Title: Time trend of global uterine cancer burden: An age-period-cohort analysis from 1990 to 2019 and predictions in a 25-year period

Figure S1 Counts and age-standardized rates of uterine cancer incidence and death at the global level, 1990 to 2019

Figure S2 Age patterns of incidence and deaths of uterine cancer by SDI in 2019.

Figure S3 Age-standardized rates of uterine cancer globally and for 21 regions by SDI, 1990 to 2019

Table S1 Age-standardized death rate and its change trends of uterine cancer, 1990 to 2019.

Table S2 The incidence information of uterine cancer in 1990 and 2019 among all countries/territories.

Table S3 The death information of uterine cancer in 1990 and 2019 among all countries/territories.

Figure S1 Counts and age-standardized rates of uterine cancer incidence and death at the global level, 1990 to 2019

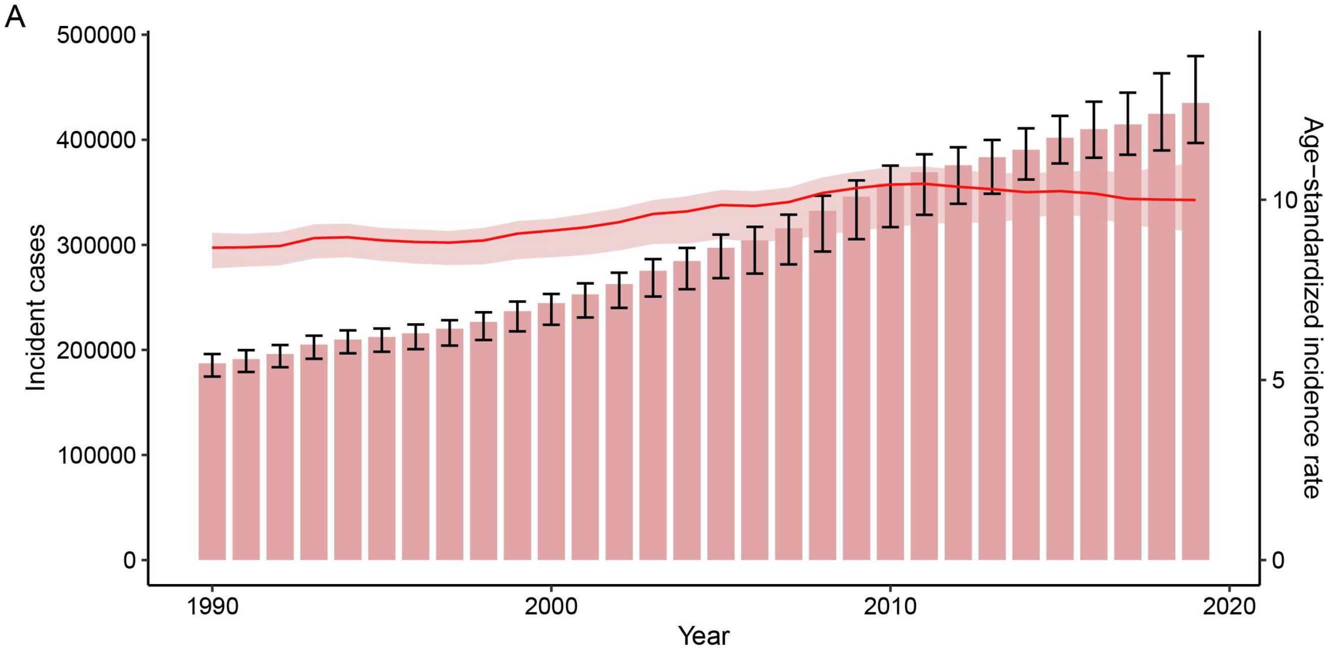

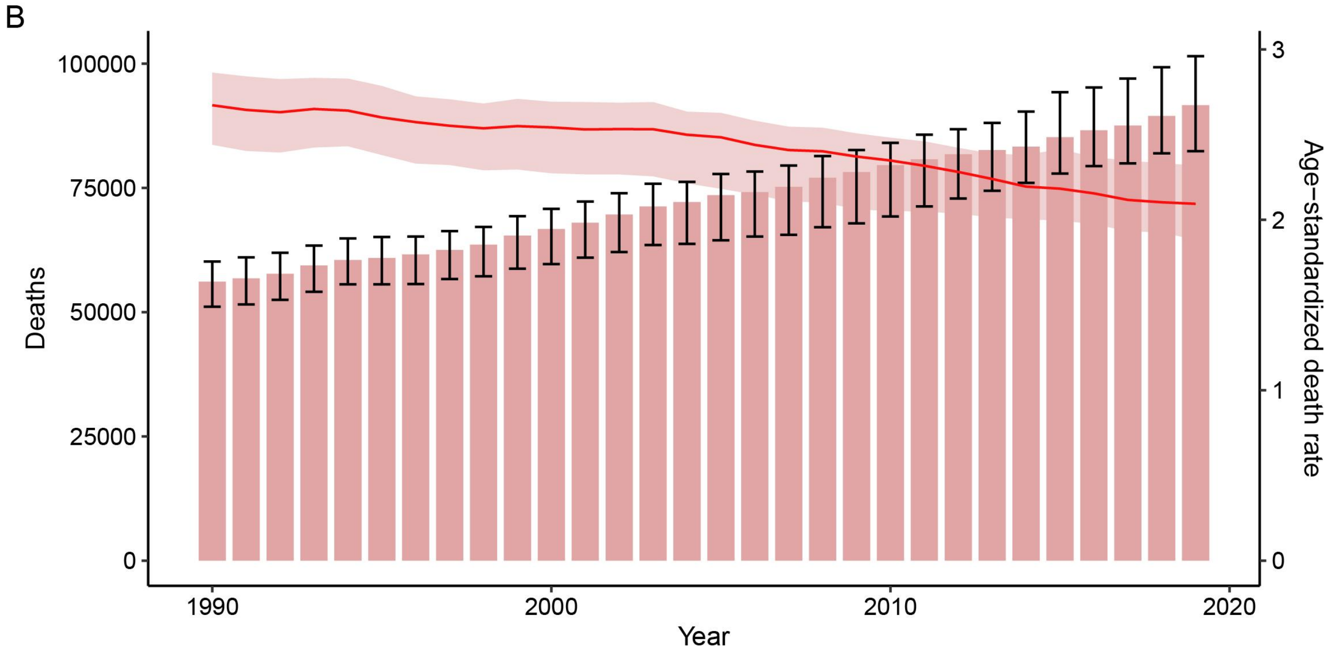

Figure S2 Age patterns of incidence and deaths of uterine cancer by SDI in 2019.

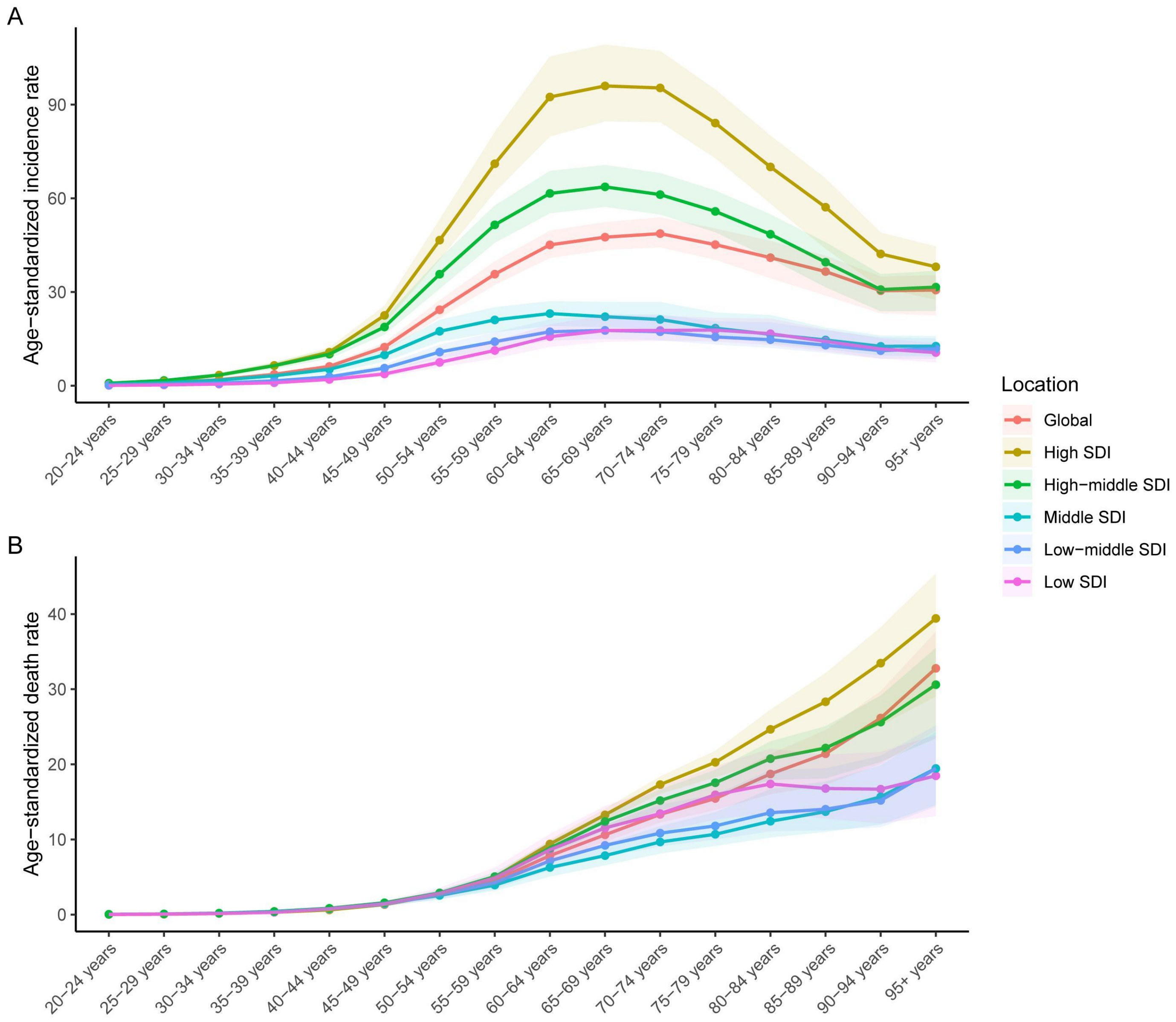

Figure S3 Age-standardized rates of uterine cancer globally and for 21 regions by SDI, 1990 to 2019

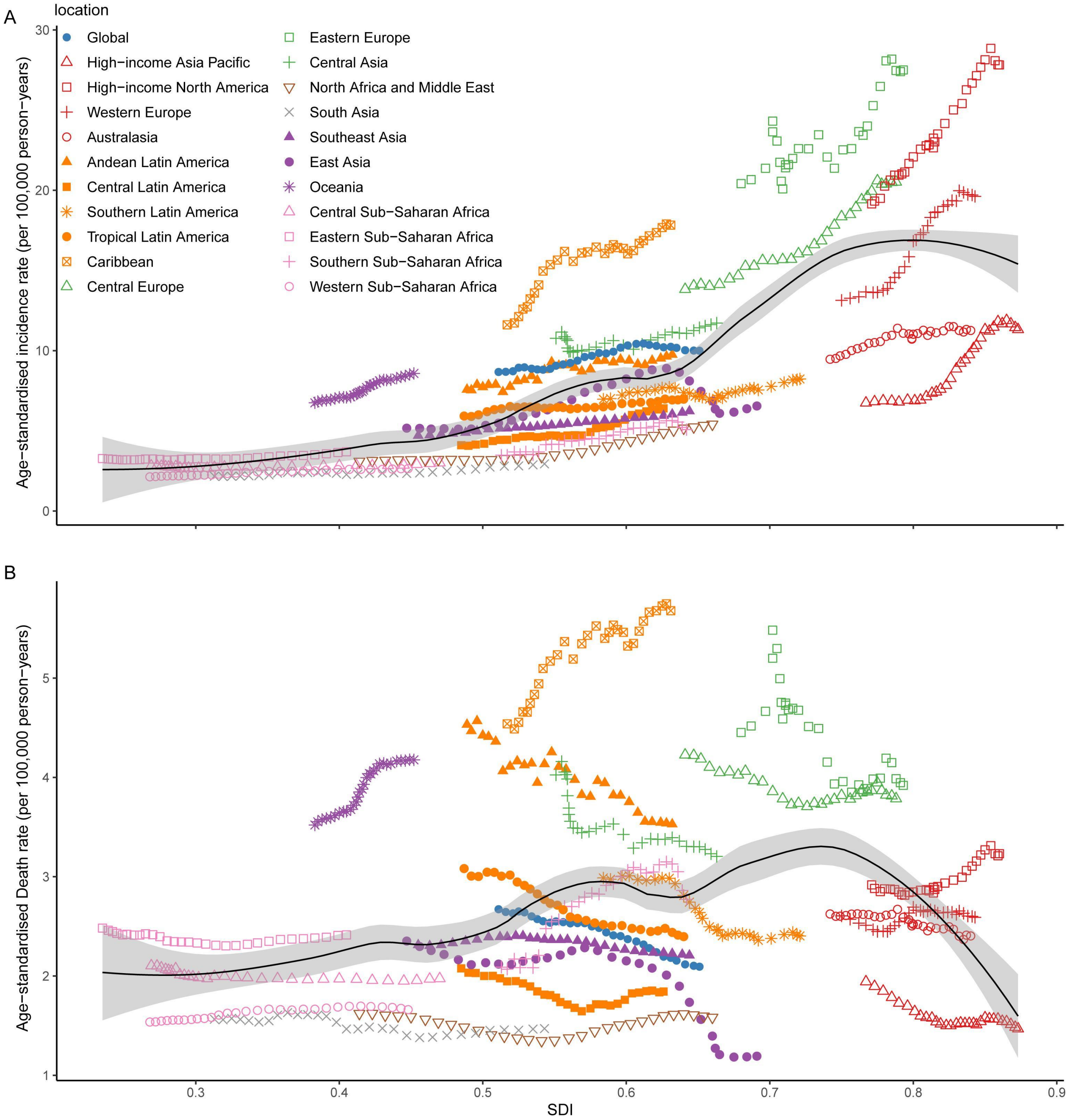

Table S1 Age-standardized death rate and its change trends of uterine cancer, 1990 to 2019.

	ASIR in 1990	ASIR in 2019	Percentage change of rate, 1990 to 2019 (%)
Global	2.67 (2.44-2.86)	2.09 (1.88-2.32)	-21.6 (-26.8 to -14.7)
Different SDI			
High SDI	2.73 (2.57-2.81)	2.52 (2.32-2.64)	-7.6 (-11.1 to -3.6)
High-middle SDI	3.23 (3.03-3.42)	2.33 (2.12-2.55)	-27.7 (-33.1 to -20.8)
Middle SDI	2.22 (1.77-2.52)	1.61 (1.36-1.87)	-27.2 (-37.2 to -12.1)
Low-middle SDI	1.93 (1.61-2.36)	1.75 (1.49-2.21)	-9.5 (-22.3 to 5.5)
Low SDI	2.08 (1.64-2.65)	2.1 (1.72-2.63)	0.9 (-15.5 to 23.8)
GBD Region			
Andean Latin America	4.53 (3.67-5.21)	3.53 (2.8-4.63)	-22.1 (-38.1 to 0.1)
Australasia	2.62 (2.46-2.77)	2.4 (2.13-2.65)	-8.4 (-17.4 to 0.7)
Caribbean	4.54 (4.18-4.99)	5.68 (4.86-6.62)	25.1 (5.9 to 45.1)
Central Asia	4.02 (3.8-4.26)	3.2 (2.88-3.57)	-20.4 (-28.5 to -11.4)
Central Europe	4.23 (4.05-4.47)	3.79 (3.28-4.36)	-10.4 (-21.6 to 2.3)
Central Latin America	2.08 (1.97-2.17)	1.84 (1.57-2.16)	-11.2 (-23.5 to 3.4)
Central Sub-Saharan Africa	2.1 (1.59-2.93)	1.97 (1.42-2.8)	-6.2 (-32.1 to 27.2)
East Asia	2.35 (1.8-2.86)	1.19 (0.93-1.65)	-49.3 (-61.7 to -27.2)
Eastern Europe	4.45 (4.27-4.64)	3.92 (3.39-4.51)	-12 (-23.3 to 0.6)
Eastern Sub-Saharan Africa	2.48 (1.78-3.09)	2.41 (1.7-3.01)	-2.8 (-19.2 to 19.4)
High-income Asia Pacific	1.94 (1.71-2.04)	1.47 (1.3-1.58)	-24.4 (-30.5 to -13.5)
High-income North America	2.92 (2.76-3.01)	3.23 (3.03-3.38)	10.8 (6.8 to 14.8)
North Africa and Middle East	1.62 (1.25-1.99)	1.58 (1.17-1.85)	-2.4 (-24.7 to 28.8)
Oceania	3.52 (2.45-4.39)	4.18 (2.44-5.5)	18.6 (-12 to 55.8)
South Asia	1.57 (1.23-2.02)	1.47 (1.16-1.87)	-6.1 (-25.8 to 18.2)

Southeast Asia	2.31 (1.73-2.73)	2.21 (1.6-2.6)	-4.5 (-20.9 to 13.6)
Southern Latin America	2.99 (2.82-3.15)	2.4 (2.19-2.62)	-19.5 (-26.6 to -11.2)
Southern Sub-Saharan Africa	2.09 (1.65-2.48)	2.77 (1.92-3.18)	32.7 (7.3 to 55.1)
Tropical Latin America	3.08 (2.89-3.22)	2.4 (2.2-2.57)	-22.2 (-27.1 to -16.7)
Western Europe	2.61 (2.46-2.69)	2.59 (2.35-2.74)	-0.5 (-5.4 to 3.8)
Western Sub-Saharan Africa	1.54 (1.24-2.32)	1.66 (1.36-2.27)	8.1 (-12 to 30.7)

ASDR: age-standardized death rate; SDI: socio-demographic index.

Table S2 The incidence information of uterine cancer in 1990 and 2019 among all countries/territories.

Country/region	Incident cases in 1990	ASIR in 1990	Incident cases in 2019	ASIR in 2019
Afghanistan	89 (45-179)	2.38 (1.25-4.8)	241 (127-408)	3.06 (1.75-5.46)
Albania	78 (68-101)	6.65 (5.8-9.12)	241 (175-344)	12.16 (8.55-16.97)
Algeria	121 (84-157)	1.94 (1.31-2.47)	400 (296-525)	2.26 (1.67-2.96)
American Samoa	2 (2-3)	17.59 (13.37-26.8)	8 (5-10)	29.82 (19.3-39.04)
Andorra	5 (3-7)	16.79 (11.82-24.69)	15 (10-21)	22.39 (15.29-31.94)
Angola	56 (37-85)	2.64 (1.71-3.91)	206 (135-312)	3.11 (2.07-4.6)
Antigua and Barbuda	2 (2-3)	8.19 (7.11-9.44)	9 (7-11)	16.23 (13.27-19.52)
Argentina	1392 (1300-1487)	7.88 (7.37-8.41)	2382 (1829-3027)	8.36 (6.35-10.66)
Armenia	149 (135-164)	9.08 (8.24-9.96)	362 (294-439)	15.81 (12.87-19.12)
Australia	919 (850-978)	9.04 (8.35-9.64)	2085 (1608-2678)	10.35 (7.91-13.33)
Austria	1165 (1067-1265)	18.68 (17.07-20.33)	1412 (1129-1759)	16.62 (13.07-20.88)
Azerbaijan	243 (191-289)	7.85 (6.17-9.29)	573 (410-870)	9.72 (7.06-14.79)
Bahamas	12 (10-13)	12.39 (11.09-13.86)	34 (27-42)	15.2 (12.2-18.87)
Bahrain	5 (3-7)	5.22 (3.9-7.38)	44 (27-59)	9.13 (6.15-11.74)
Bangladesh	402 (272-680)	1.83 (1.25-3.07)	1246 (832-2600)	1.9 (1.27-3.99)
Barbados	21 (19-23)	14.01 (12.61-15.59)	62 (50-75)	24.28 (19.58-29.57)
Belarus	974 (881-1073)	12.34 (11.15-13.53)	1296 (969-1714)	14.37 (10.71-19.07)
Belgium	1099 (1014-1193)	13.73 (12.67-14.88)	1954 (1496-2499)	18.13 (13.76-23.43)
Belize	4 (3-5)	8.03 (6.95-9.75)	18 (15-21)	11.83 (10-13.7)
Benin	30 (24-40)	2.94 (2.29-3.84)	92 (67-127)	3.53 (2.59-4.87)
Bermuda	5 (4-6)	14.32 (12.43-16.48)	10 (8-13)	15.51 (12.17-19.92)
Bhutan	3 (2-5)	2.04 (1.18-3.79)	9 (5-19)	3.05 (1.77-6.71)
Bolivia (Plurinational State of)	137 (102-187)	7.7 (5.78-10.59)	517 (379-746)	10.86 (8.03-15.63)

Bosnia and Herzegovina	169 (145-242)	6.82 (5.9-9.98)	472 (329-616)	15.71 (10.57-20.68)
Botswana	14 (10-20)	4.25 (3.01-6.03)	63 (39-95)	7.77 (4.94-11.49)
Brazil	2871 (2729-3006)	5.87 (5.57-6.15)	9081 (8481-9717)	6.92 (6.47-7.4)
Brunei Darussalam	5 (4-6)	7.8 (5.62-9.86)	17 (11-23)	8.62 (5.78-10.95)
Bulgaria	1365 (1243-1492)	21.16 (19.31-23.1)	2003 (1543-2543)	30.66 (23.26-39.76)
Burkina Faso	68 (49-93)	2.9 (2.12-3.94)	182 (131-249)	3.59 (2.61-4.85)
Burundi	51 (30-70)	3.87 (2.26-5.31)	82 (49-119)	3.53 (2.13-5.11)
Cabo Verde	6 (5-7)	4.54 (3.56-5.64)	17 (13-23)	7.16 (5.53-9.59)
Cambodia	155 (89-234)	5.36 (3.21-8.07)	542 (322-736)	7.2 (4.39-9.68)
Cameroon	91 (58-128)	3.89 (2.47-5.46)	280 (179-412)	4.47 (2.92-6.47)
Canada	2514 (2347-2687)	14.53 (13.56-15.54)	6575 (5029-8406)	19.56 (14.82-25.07)
Central African Republic	21 (15-30)	3.09 (2.22-4.37)	36 (23-57)	2.86 (1.81-4.49)
Chad	38 (28-52)	2.6 (1.91-3.6)	84 (62-116)	3.27 (2.41-4.48)
Chile	222 (202-243)	4.04 (3.68-4.41)	1037 (792-1334)	7.96 (6.08-10.26)
China	24283 (18182-29960)	5.13 (3.89-6.29)	66744 (51379-92052)	6.39 (4.89-8.74)
Colombia	390 (360-419)	4.03 (3.73-4.34)	1719 (1291-2226)	6.01 (4.52-7.8)
Comoros	4 (2-6)	3.7 (2.01-5.16)	12 (8-17)	4.51 (2.82-6.24)
Congo	27 (19-38)	4.2 (2.96-5.92)	66 (45-99)	4.53 (3.13-6.62)
Cook Islands	1 (0-1)	7.81 (5.54-10.99)	1 (1-1)	8.08 (5.61-11.01)
Costa Rica	48 (43-53)	5.14 (4.6-5.74)	289 (217-379)	10.25 (7.73-13.43)
C 么 te d'Ivoire	58 (42-80)	3.05 (2.22-4.13)	174 (123-241)	3.41 (2.46-4.59)
Croatia	713 (624-800)	19.02 (16.7-21.3)	1222 (926-1572)	27.85 (20.93-36.12)
Cuba	860 (789-931)	16.49 (15.11-17.88)	2469 (1987-3090)	26.49 (21.22-33.22)
Cyprus	52 (40-68)	12.23 (9.44-15.79)	203 (115-255)	20.54 (11.63-25.88)
Czechia	1511 (1411-1611)	19.92 (18.55-21.28)	2166 (1718-2692)	21.21 (16.78-26.38)
Democratic People's Republic of	863 (487-1249)	7.92 (4.56-11.41)	1308 (701-1915)	7.37 (3.89-10.87)

Korea				
Democratic Republic of the Congo	251 (181-362)	2.71 (1.98-3.92)	576 (385-866)	2.82 (1.88-4.24)
Denmark	699 (651-754)	17.55 (16.28-18.96)	973 (750-1232)	17.7 (13.61-22.53)
Djibouti	3 (2-4)	3.68 (2.17-5.14)	14 (8-22)	4.91 (2.93-7.36)
Dominica	3 (3-4)	8.78 (7.1-11.98)	5 (3-6)	10.54 (7.85-13.91)
Dominican Republic	159 (119-190)	7.58 (5.77-9.05)	688 (434-969)	13.79 (8.7-19.38)
Ecuador	270 (168-307)	9.54 (5.92-10.85)	871 (637-1205)	10.8 (7.89-14.88)
Egypt	390 (278-454)	2.4 (1.81-2.79)	1493 (939-2120)	4.45 (2.91-6.24)
El Salvador	83 (64-95)	5.1 (3.91-5.86)	238 (169-353)	7.13 (5.05-10.59)
Equatorial Guinea	3 (2-5)	2.5 (1.5-3.91)	13 (7-21)	4.34 (2.61-6.91)
Eritrea	20 (12-30)	3.27 (1.98-4.97)	78 (47-112)	4.91 (2.93-6.93)
Estonia	192 (173-213)	15.33 (13.82-17)	334 (251-434)	25.08 (18.71-32.97)
Eswatini	7 (5-9)	4.23 (3.13-5.69)	18 (11-28)	5.31 (3.14-8.05)
Ethiopia	347 (213-529)	3.35 (2.01-5.01)	590 (424-874)	2.84 (2.06-4.23)
Fiji	20 (13-27)	9.29 (6.17-12.42)	47 (25-65)	10.96 (5.86-14.84)
Finland	533 (496-574)	13.43 (12.46-14.54)	1084 (836-1387)	18.47 (14.12-23.89)
France	5736 (5289-6161)	13.45 (12.47-14.42)	11768 (9035-15100)	18.67 (14.27-24.18)
Gabon	13 (9-18)	3.93 (2.75-5.63)	26 (17-38)	4.47 (2.99-6.56)
Gambia	3 (2-5)	1.98 (1.39-2.75)	15 (10-21)	2.97 (2.04-4.16)
Georgia	856 (746-981)	23.49 (20.51-26.88)	706 (561-870)	23.68 (18.78-29.09)
Germany	10360 (9661-11070)	14.64 (13.68-15.6)	13823 (10739-17926)	15.52 (11.94-20.16)
Ghana	143 (68-194)	4.16 (1.99-5.57)	463 (225-639)	4.93 (2.41-6.77)
Greece	1065 (980-1152)	13.78 (12.67-14.86)	2034 (1571-2598)	20.6 (15.79-26.46)
Greenland	1 (1-1)	3.66 (2.93-4.94)	1 (1-2)	3.86 (2.7-5.04)
Grenada	6 (5-7)	16.46 (14.33-18.82)	17 (15-20)	29.41 (25.01-33.96)
Guam	5 (4-6)	11.51 (9.17-14.57)	11 (8-14)	11.53 (8.11-14.51)

Guatemala	101 (86-118)	5.17 (4.45-5.96)	411 (314-526)	6.53 (4.99-8.34)
Guinea	54 (41-76)	3.19 (2.41-4.5)	103 (73-143)	3.65 (2.6-5.13)
Guinea-Bissau	8 (5-11)	3.67 (2.49-5.27)	17 (12-24)	4.24 (2.99-6.03)
Guyana	22 (18-27)	10.4 (8.61-12.3)	53 (40-69)	14.79 (11.29-19.04)
Haiti	159 (109-221)	8.58 (6.04-12.44)	375 (246-549)	9.03 (5.94-13.08)
Honduras	61 (45-95)	5.02 (3.71-8.28)	356 (202-588)	10.23 (5.89-17.36)
Hungary	1349 (1254-1444)	16.65 (15.47-17.81)	1717 (1366-2111)	17.56 (13.93-21.73)
Iceland	22 (19-25)	15.47 (13.77-17.58)	35 (28-42)	13.35 (11-16.07)
India	4228 (3063-5489)	1.88 (1.36-2.41)	15262 (10843-19608)	2.53 (1.81-3.26)
Indonesia	2979 (1899-3713)	5.05 (3.31-6.25)	8731 (5030-11787)	6.85 (4.09-9.16)
Iran (Islamic Republic of)	305 (182-392)	2.12 (1.33-2.71)	1628 (760-1950)	4.02 (1.95-4.79)
Iraq	128 (82-240)	3.02 (1.93-5.71)	932 (629-1298)	6.74 (4.71-9.28)
Ireland	265 (238-293)	12.81 (11.52-14.22)	776 (578-1006)	21.12 (15.66-27.53)
Israel	216 (193-239)	8.39 (7.53-9.26)	924 (704-1183)	15.71 (11.98-20.16)
Italy	4159 (3941-4357)	8.92 (8.47-9.36)	16899 (13088-21487)	26.93 (20.7-34.54)
Jamaica	64 (56-71)	6.93 (6.12-7.78)	280 (215-356)	18.5 (14.2-23.53)
Japan	6015 (5719-6261)	6.7 (6.39-6.98)	15543 (12190-19293)	14.51 (11.4-18.21)
Jordan	40 (30-57)	5.57 (4.17-8)	255 (189-334)	7.35 (5.49-9.57)
Kazakhstan	1083 (997-1176)	13.65 (12.6-14.83)	1434 (1196-1700)	13.41 (11.25-15.83)
Kenya	73 (54-102)	1.65 (1.23-2.31)	278 (208-399)	2.24 (1.7-3.19)
Kiribati	2 (1-3)	8.56 (3.96-11.62)	4 (2-5)	8.52 (3.66-11.79)
Kuwait	17 (14-19)	6.28 (5.3-7.41)	129 (102-164)	10.03 (7.83-12.8)
Kyrgyzstan	129 (114-145)	7.15 (6.34-8.02)	271 (226-326)	9.43 (7.89-11.34)
Lao People's Democratic Republic	72 (40-110)	5.91 (3.42-8.85)	161 (91-223)	6.17 (3.68-8.53)
Latvia	347 (316-380)	15.89 (14.45-17.41)	602 (453-792)	29.1 (21.51-39.1)
Lebanon	82 (62-106)	6.84 (5.22-8.83)	358 (253-485)	12.62 (8.89-17.21)

Lesotho	16 (12-21)	2.83 (2.15-3.81)	44 (26-66)	5.76 (3.53-8.58)
Liberia	17 (13-23)	3.24 (2.45-4.35)	37 (25-54)	3.71 (2.46-5.35)
Libya	37 (25-51)	4.05 (2.71-5.51)	163 (109-227)	5.91 (3.93-8.14)
Lithuania	317 (293-343)	11.96 (11.05-12.97)	441 (349-550)	15.13 (11.8-18.98)
Luxembourg	67 (60-74)	22.75 (20.28-25.27)	105 (82-131)	21.75 (17.04-26.98)
Madagascar	88 (53-110)	3.24 (1.96-4.03)	218 (131-312)	3.58 (2.13-5.05)
Malawi	48 (35-61)	2.22 (1.64-2.83)	88 (61-119)	2.14 (1.51-2.86)
Malaysia	303 (223-372)	5.95 (4.37-7.27)	1310 (876-1749)	9.14 (6.14-12.19)
Maldives	2 (1-3)	4.76 (2.37-7.05)	8 (6-10)	4.87 (3.7-6.28)
Mali	52 (41-68)	2.4 (1.87-3.18)	117 (84-162)	2.68 (1.99-3.71)
Malta	37 (32-41)	15.57 (13.7-17.61)	75 (59-93)	17.32 (13.82-21.6)
Marshall Islands	1 (1-1)	9.65 (6.15-12.74)	3 (1-4)	13.6 (6.89-20.04)
Mauritania	23 (17-31)	4.47 (3.17-5.97)	48 (33-67)	4.6 (3.13-6.33)
Mauritius	57 (51-62)	13.65 (12.32-15.02)	117 (92-145)	12.25 (9.73-15.25)
Mexico	745 (722-766)	3.15 (3.04-3.25)	3550 (2870-4333)	5.4 (4.37-6.58)
Micronesia (Federated States of)	3 (2-4)	11.27 (6.8-15.97)	7 (3-11)	16.2 (8.12-24.77)
Monaco	2 (2-3)	7.33 (5.2-10.05)	4 (3-5)	9.93 (6.69-13.1)
Mongolia	33 (24-45)	4.94 (3.64-6.7)	109 (75-154)	6.72 (4.65-9.35)
Montenegro	40 (33-54)	11.45 (9.38-15.32)	86 (69-114)	17.24 (13.77-22.47)
Morocco	180 (135-229)	2.46 (1.85-3.14)	759 (520-1027)	4.44 (3.06-5.92)
Mozambique	100 (60-143)	3.1 (1.85-4.4)	255 (151-382)	4.07 (2.41-6.07)
Myanmar	754 (433-1174)	5.51 (3.28-8.5)	1710 (1091-2290)	6.02 (3.97-8)
Namibia	12 (9-17)	3.11 (2.29-4.26)	36 (24-51)	4.36 (2.91-6.18)
Nauru	0 (0-1)	16.89 (9.36-23.87)	1 (0-1)	19.9 (8.88-28.49)
Nepal	90 (55-161)	1.83 (1.12-3.32)	298 (192-654)	2.46 (1.57-5.38)
Netherlands	1543 (1428-1655)	14.8 (13.67-15.9)	3761 (2868-4792)	23.02 (17.48-29.53)

New Zealand	236 (214-258)	11.6 (10.53-12.71)	603 (454-775)	15.93 (11.95-20.56)
Nicaragua	20 (16-25)	2.2 (1.8-2.85)	133 (98-173)	5.12 (3.8-6.72)
Niger	36 (24-51)	2.66 (1.79-3.78)	123 (89-172)	3.02 (2.19-4.18)
Nigeria	206 (122-472)	0.98 (0.58-2.25)	631 (377-1367)	1.31 (0.8-2.83)
Niue	0 (0-0)	12.89 (7.47-18.33)	0 (0-0)	17.92 (8.54-25.82)
North Macedonia	118 (99-168)	11.37 (9.58-16.42)	410 (289-533)	25.12 (17.39-32.84)
Northern Mariana Islands	2 (1-4)	21.24 (14.23-40.19)	10 (7-13)	32.77 (21.3-42.36)
Norway	436 (409-463)	13.55 (12.73-14.39)	839 (666-1035)	18.62 (14.71-23.08)
Oman	6 (4-9)	2.04 (1.38-2.94)	31 (23-41)	3.85 (2.83-4.95)
Pakistan	1299 (1042-1738)	4.9 (3.88-6.62)	5011 (3575-7055)	8.6 (6.21-11.9)
Palau	0 (0-0)	1.79 (1.25-2.54)	0 (0-0)	2 (1.43-2.78)
Palestine	40 (26-58)	7.91 (5.27-11.42)	169 (98-216)	12.54 (7.41-15.88)
Panama	30 (27-34)	3.99 (3.55-4.5)	206 (155-272)	9.74 (7.35-12.92)
Papua New Guinea	51 (32-71)	4.98 (3.21-6.81)	179 (94-262)	6.66 (3.62-9.64)
Paraguay	97 (66-118)	8.18 (5.56-9.83)	267 (186-419)	9.02 (6.33-14.23)
Peru	434 (348-534)	6.68 (5.39-8.17)	1514 (1078-2087)	8.98 (6.42-12.34)
Philippines	1225 (853-1446)	6.72 (4.99-8.01)	3331 (2297-4501)	7.12 (5.04-9.59)
Poland	2955 (2826-3091)	11.97 (11.46-12.5)	7610 (5898-9610)	20.9 (16.31-26.42)
Portugal	1080 (984-1179)	14.59 (13.28-15.96)	2166 (1676-2808)	19.48 (14.99-25.16)
Puerto Rico	164 (149-181)	8.52 (7.72-9.39)	429 (327-554)	13.38 (10.1-17.42)
Qatar	2 (1-2)	4.03 (2.01-5.56)	23 (11-32)	9.49 (3.85-13.29)
Republic of Korea	1350 (577-1586)	6.89 (3.03-8.06)	2035 (1335-2625)	4.64 (3.02-6.06)
Republic of Moldova	302 (276-327)	11.32 (10.36-12.25)	458 (384-543)	14.33 (12-17.02)
Romania	1538 (1431-1658)	10.14 (9.46-10.9)	2731 (2187-3368)	15.63 (12.38-19.41)
Russian Federation	27140 (26082-28512)	24.65 (23.59-26)	42711 (35106-51779)	32.55 (26.58-39.65)
Rwanda	73 (43-98)	4.29 (2.57-5.69)	146 (97-197)	4.06 (2.72-5.34)

Saint Kitts and Nevis	4 (3-4)	20.01 (17.74-22.63)	8 (6-10)	20.83 (16.02-26.3)
Saint Lucia	5 (4-5)	10.26 (9.24-11.32)	15 (12-18)	13.07 (10.83-15.74)
Saint Vincent and the Grenadines	6 (5-6)	14.44 (12.9-16)	13 (11-15)	19.67 (16.84-22.95)
Samoa	5 (3-6)	9.79 (7.18-13.49)	9 (6-12)	11.47 (8.02-15.77)
San Marino	1 (1-1)	4.33 (3.36-5.4)	2 (1-3)	5.83 (4.01-8.67)
Sao Tome and Principe	2 (1-2)	5.19 (3.89-6.63)	5 (3-7)	8.36 (5.47-11.6)
Saudi Arabia	54 (38-81)	2.07 (1.45-3.04)	641 (439-878)	6.73 (4.57-9.02)
Senegal	50 (37-67)	3.07 (2.29-4.18)	148 (106-201)	3.77 (2.74-5.07)
Serbia	724 (583-978)	11.55 (9.35-15.62)	1768 (1199-2357)	23.04 (15.4-30.59)
Seychelles	2 (2-3)	7.53 (6.06-9.4)	6 (5-8)	10.32 (8.05-12.92)
Sierra Leone	23 (17-32)	2.53 (1.85-3.43)	65 (43-90)	3.6 (2.42-5.03)
Singapore	49 (44-55)	3.79 (3.38-4.22)	403 (313-512)	9.93 (7.73-12.59)
Slovakia	558 (455-649)	16.88 (13.79-19.7)	1058 (739-1410)	22.01 (15.39-29.56)
Slovenia	219 (167-286)	15.48 (11.74-20.3)	376 (279-501)	18.29 (13.55-24.75)
Solomon Islands	9 (5-14)	11.74 (7.33-17.24)	34 (17-52)	17.34 (9.1-25.3)
Somalia	49 (28-74)	3.35 (1.93-4.98)	128 (68-212)	3.27 (1.77-5.4)
South Africa	373 (306-446)	3.03 (2.45-3.65)	1132 (824-1350)	4.36 (3.14-5.19)
South Sudan	38 (22-56)	3.54 (2.05-5.28)	61 (38-92)	3.28 (2.05-4.86)
Spain	4218 (3910-4535)	14.84 (13.78-15.97)	9752 (7443-12370)	22.01 (16.66-27.99)
Sri Lanka	202 (164-301)	3.47 (2.83-5.14)	935 (619-1320)	6.53 (4.33-9.22)
Sudan	66 (42-115)	1.39 (0.9-2.43)	227 (142-364)	2.25 (1.46-3.63)
Suriname	6 (5-8)	4.14 (3.41-5.46)	20 (14-25)	6.07 (4.37-7.73)
Sweden	1177 (1087-1274)	16.27 (15.03-17.72)	1739 (1395-2166)	17.85 (14.28-22.39)
Switzerland	734 (668-807)	13.73 (12.45-15.14)	1111 (856-1410)	13.51 (10.32-17.3)
Syrian Arab Republic	79 (56-108)	2.64 (1.91-3.92)	261 (176-394)	3.81 (2.59-5.89)
Taiwan (Province of China)	299 (274-323)	3.73 (3.42-4.03)	2865 (2169-3796)	14.49 (10.93-19.25)

Tajikistan	98 (81-130)	6.02 (5.02-8.06)	354 (221-468)	10.45 (6.91-13.54)
Thailand	818 (576-1005)	3.8 (2.75-4.64)	2829 (1784-3946)	5.06 (3.19-7.03)
Timor-Leste	8 (5-11)	4.6 (2.81-6.55)	26 (15-36)	6 (3.62-8.21)
Togo	22 (17-29)	3.3 (2.54-4.27)	75 (52-106)	3.57 (2.47-4.97)
Tokelau	0 (0-0)	12.19 (7.13-17.25)	0 (0-0)	17.08 (8.12-24.45)
Tonga	2 (1-3)	7.78 (4.68-11.14)	4 (2-6)	9.25 (4.55-14.67)
Trinidad and Tobago	49 (45-54)	11.16 (10.21-12.28)	157 (117-206)	16.02 (11.9-20.96)
Tunisia	67 (51-85)	2.62 (1.98-3.31)	297 (204-426)	4.43 (3.04-6.33)
Turkey	1087 (679-1437)	5.49 (3.47-7.23)	4213 (2445-5546)	8.88 (5.18-11.65)
Turkmenistan	68 (62-74)	5.73 (5.2-6.26)	76 (58-100)	3.15 (2.43-4.1)
Tuvalu	0 (0-1)	11.15 (6.82-15.67)	1 (0-1)	13.51 (6.66-19.59)
Uganda	118 (81-175)	3.49 (2.46-5.16)	521 (378-680)	6.42 (4.73-8.33)
Ukraine	5300 (4773-5872)	12.17 (11-13.42)	6671 (5101-8618)	16.19 (12.26-20.86)
United Arab Emirates	7 (3-13)	3.92 (1.51-7.99)	80 (36-130)	4.72 (1.91-8.42)
United Kingdom	5330 (5120-5467)	11.52 (11.14-11.81)	10973 (8705-13770)	18.41 (14.52-23.19)
United Republic of Tanzania	218 (133-287)	3.78 (2.27-4.97)	603 (388-786)	4.56 (2.94-5.85)
United States of America	33309 (31987-34256)	19.63 (18.96-20.17)	80070 (65805-96681)	28.8 (23.5-34.93)
United States Virgin Islands	5 (4-7)	10.72 (8.49-13.68)	12 (9-15)	12.02 (8.98-15.48)
Uruguay	159 (144-175)	7.6 (6.89-8.35)	226 (175-288)	8.1 (6.2-10.37)
Uzbekistan	393 (358-430)	5.78 (5.25-6.32)	1571 (1265-1923)	10.22 (8.34-12.42)
Vanuatu	2 (1-4)	7.1 (3.93-10.71)	9 (4-14)	9.56 (4.57-14.59)
Venezuela (Bolivarian Republic of)	415 (385-446)	7.74 (7.17-8.34)	1466 (1077-1968)	9.23 (6.8-12.36)
Viet Nam	551 (410-718)	2.36 (1.76-3.03)	2401 (1510-3332)	4.31 (2.73-5.87)
Yemen	37 (22-69)	1.37 (0.82-2.66)	159 (107-252)	2.06 (1.4-3.38)
Zambia	69 (42-91)	4.66 (2.83-6.06)	195 (123-275)	5.23 (3.29-7.28)
Zimbabwe	132 (99-165)	6.08 (4.63-7.58)	383 (214-530)	8.96 (5.2-12.32)

ASIR: age-standardized incident rate

Table S3 The death information of uterine cancer in 1990 and 2019 among all countries/territories.

Country/region	Deaths in 1990	ASDR in 1990	Deaths in 2019	ASDR in 2019
Afghanistan	61 (32-125)	1.73 (0.95-3.58)	127 (72-223)	1.87 (1.12-3.44)
Albania	26 (22-41)	2.36 (2.01-3.92)	44 (31-76)	2.02 (1.43-3.31)
Algeria	56 (36-72)	1.09 (0.66-1.38)	110 (80-143)	0.75 (0.54-0.97)
American Samoa	1 (1-1)	7.15 (5.49-11.01)	3 (2-3)	10.74 (7.18-13.86)
Andorra	1 (1-1)	3.14 (2.26-4.62)	2 (1-3)	2.84 (1.96-4.03)
Angola	40 (26-58)	2.05 (1.3-3)	115 (76-171)	1.99 (1.34-2.85)
Antigua and Barbuda	1 (1-1)	3.1 (2.7-3.57)	3 (2-3)	5.43 (4.49-6.45)
Argentina	603 (563-642)	3.34 (3.12-3.55)	797 (709-892)	2.58 (2.3-2.88)
Armenia	51 (47-56)	3.3 (3.02-3.59)	89 (74-107)	3.75 (3.1-4.45)
Australia	279 (258-296)	2.54 (2.36-2.69)	524 (455-584)	2.28 (2-2.54)
Austria	252 (232-269)	3.39 (3.13-3.62)	231 (201-259)	2.17 (1.92-2.42)
Azerbaijan	99 (78-116)	3.37 (2.65-3.96)	153 (111-236)	3.02 (2.18-4.69)
Bahamas	4 (4-5)	4.75 (4.27-5.29)	11 (9-14)	5.24 (4.3-6.37)
Bahrain	2 (1-3)	2.42 (1.86-3.44)	8 (5-10)	2.28 (1.68-2.87)
Bangladesh	271 (184-456)	1.34 (0.91-2.26)	562 (377-1176)	0.93 (0.63-1.95)
Barbados	9 (8-10)	5.26 (4.78-5.83)	20 (17-24)	7.55 (6.22-9.01)
Belarus	264 (240-286)	3.19 (2.91-3.45)	224 (172-288)	2.24 (1.72-2.88)
Belgium	269 (248-291)	2.9 (2.69-3.13)	371 (318-416)	2.7 (2.37-3.02)
Belize	2 (2-2)	3.92 (3.41-4.58)	6 (5-7)	4.63 (3.98-5.31)
Benin	22 (17-29)	2.2 (1.71-2.93)	56 (41-76)	2.33 (1.72-3.14)
Bermuda	2 (1-2)	4.67 (4.09-5.26)	2 (2-3)	3.17 (2.48-4.04)
Bhutan	2 (1-3)	1.5 (0.89-2.78)	4 (2-8)	1.46 (0.85-3.25)
Bolivia (Plurinational State of)	92 (69-125)	5.48 (4.14-7.52)	242 (184-344)	5.4 (4.12-7.62)
Bosnia and Herzegovina	60 (52-91)	2.64 (2.27-4.09)	109 (81-141)	3.3 (2.42-4.25)

Botswana	8 (6-12)	2.76 (1.98-3.83)	26 (17-38)	3.67 (2.35-5.28)
Brazil	1374 (1300-1436)	3.06 (2.87-3.2)	3093 (2830-3309)	2.38 (2.17-2.55)
Brunei Darussalam	2 (1-2)	3.3 (2.44-4.14)	5 (3-6)	2.81 (2.09-3.49)
Bulgaria	300 (277-325)	4.51 (4.17-4.86)	395 (312-496)	5.1 (4-6.45)
Burkina Faso	49 (35-66)	2.22 (1.63-3.02)	110 (80-147)	2.38 (1.76-3.12)
Burundi	37 (22-51)	2.97 (1.75-4.04)	50 (30-72)	2.44 (1.45-3.54)
Cabo Verde	4 (3-5)	2.94 (2.25-3.63)	8 (6-10)	3.39 (2.5-4.32)
Cambodia	86 (52-129)	3.22 (2.05-4.77)	213 (136-281)	3.03 (2.02-3.93)
Cameroon	61 (39-85)	2.85 (1.77-3.97)	156 (102-224)	2.76 (1.85-3.89)
Canada	523 (486-554)	2.82 (2.63-2.99)	1003 (887-1122)	2.63 (2.33-2.93)
Central African Republic	15 (11-21)	2.37 (1.7-3.36)	25 (16-40)	2.2 (1.43-3.4)
Chad	28 (21-40)	2.02 (1.5-2.81)	56 (41-76)	2.38 (1.76-3.2)
Chile	96 (88-105)	1.83 (1.68-1.99)	267 (236-299)	1.99 (1.76-2.22)
China	10598 (7953-13036)	2.38 (1.81-2.9)	12223 (9425-17338)	1.17 (0.9-1.66)
Colombia	168 (156-180)	1.93 (1.78-2.06)	446 (344-569)	1.54 (1.19-1.96)
Comoros	3 (2-4)	2.85 (1.64-3.93)	7 (5-10)	2.88 (1.78-3.96)
Congo	18 (12-25)	3.01 (2.12-4.21)	36 (25-52)	2.75 (1.91-3.86)
Cook Islands	0 (0-0)	2.23 (1.6-3.17)	0 (0-0)	1.88 (1.34-2.48)
Costa Rica	16 (14-18)	1.84 (1.64-2.03)	68 (53-87)	2.45 (1.89-3.11)
C 么 te d'Ivoire	38 (27-50)	2.25 (1.65-3.05)	104 (75-141)	2.29 (1.67-3.04)
Croatia	136 (122-150)	3.57 (3.21-3.96)	180 (140-223)	3.36 (2.59-4.22)
Cuba	260 (241-281)	5 (4.63-5.4)	652 (536-807)	6.48 (5.3-8.06)
Cyprus	15 (12-21)	3.8 (2.91-5.08)	31 (18-38)	3.05 (1.83-3.74)
Czechia	460 (433-483)	5.56 (5.25-5.84)	445 (358-545)	3.62 (2.91-4.43)
Democratic People's Republic of Korea	223 (134-313)	2.19 (1.34-3.01)	365 (213-516)	2 (1.14-2.83)

Democratic Republic of the Congo	169 (122-247)	2.01 (1.48-2.89)	354 (237-529)	1.89 (1.26-2.83)
Denmark	183 (171-196)	3.89 (3.64-4.14)	183 (160-209)	2.76 (2.42-3.12)
Djibouti	2 (1-2)	2.7 (1.59-3.69)	7 (4-11)	2.99 (1.79-4.32)
Dominica	2 (1-2)	3.73 (3.07-5.19)	2 (2-3)	4.63 (3.51-5.98)
Dominican Republic	77 (59-92)	4.1 (3.13-4.88)	264 (175-361)	5.51 (3.67-7.51)
Ecuador	152 (94-172)	5.8 (3.63-6.58)	304 (226-409)	3.97 (2.97-5.33)
Egypt	197 (154-230)	1.36 (1.11-1.62)	449 (298-628)	1.64 (1.11-2.26)
El Salvador	44 (34-50)	2.82 (2.19-3.21)	77 (56-114)	2.25 (1.62-3.32)
Equatorial Guinea	2 (1-3)	1.98 (1.19-3.1)	6 (4-9)	2.34 (1.47-3.61)
Eritrea	15 (9-22)	2.64 (1.61-4.04)	48 (29-68)	3.39 (2.01-4.69)
Estonia	48 (44-53)	3.59 (3.28-3.92)	53 (40-67)	3.06 (2.33-3.89)
Eswatini	5 (3-6)	2.96 (2.2-3.99)	11 (7-16)	3.38 (2.07-5.03)
Ethiopia	247 (151-362)	2.61 (1.54-3.94)	347 (247-522)	1.86 (1.32-2.82)
Fiji	8 (5-10)	4.21 (2.9-5.47)	19 (10-25)	4.7 (2.55-6.23)
Finland	144 (133-154)	3.16 (2.94-3.38)	229 (198-259)	3.06 (2.69-3.41)
France	1517 (1377-1637)	3 (2.76-3.21)	2141 (1774-2438)	2.59 (2.21-2.93)
Gabon	8 (6-11)	2.61 (1.82-3.69)	13 (9-18)	2.44 (1.67-3.48)
Gambia	2 (2-3)	1.43 (1.02-1.96)	9 (6-12)	1.85 (1.28-2.59)
Georgia	262 (230-298)	7.04 (6.2-7.97)	195 (158-237)	5.8 (4.65-7.03)
Germany	2233 (2056-2377)	2.73 (2.53-2.89)	2584 (2256-2876)	2.28 (2.02-2.54)
Ghana	89 (43-120)	2.75 (1.4-3.64)	234 (116-322)	2.66 (1.37-3.6)
Greece	207 (192-223)	2.5 (2.32-2.67)	346 (307-381)	2.67 (2.41-2.92)
Greenland	0 (0-0)	1.64 (1.34-2.19)	0 (0-1)	1.31 (0.91-1.68)
Grenada	3 (3-3)	7.46 (6.54-8.47)	7 (6-8)	11.3 (9.79-12.99)
Guam	1 (1-2)	3.5 (2.75-4.45)	3 (2-4)	3.05 (2.25-3.81)
Guatemala	61 (53-70)	3.58 (3.13-4.05)	166 (130-206)	2.81 (2.21-3.47)

Guinea	40 (30-58)	2.44 (1.82-3.56)	65 (47-92)	2.49 (1.8-3.52)
Guinea-Bissau	6 (4-8)	2.79 (1.89-3.99)	11 (8-15)	2.9 (2.06-4.06)
Guyana	11 (9-13)	5.74 (4.8-6.7)	23 (18-29)	6.9 (5.36-8.74)
Haiti	106 (74-155)	6.17 (4.39-9.18)	219 (143-316)	5.8 (3.82-8.36)
Honduras	31 (23-53)	2.86 (2.07-5.06)	135 (79-233)	4.33 (2.56-7.46)
Hungary	457 (428-487)	5.25 (4.91-5.57)	387 (313-465)	3.25 (2.62-3.93)
Iceland	4 (4-4)	2.52 (2.25-2.83)	5 (4-6)	1.6 (1.35-1.86)
India	2680 (1941-3500)	1.34 (0.98-1.74)	7044 (5140-9093)	1.24 (0.91-1.59)
Indonesia	1407 (927-1757)	2.67 (1.82-3.3)	3273 (2023-4300)	2.86 (1.87-3.69)
Iran (Islamic Republic of)	102 (73-128)	0.85 (0.63-1.11)	349 (186-409)	0.98 (0.54-1.15)
Iraq	57 (37-113)	1.45 (0.93-2.92)	230 (166-311)	1.92 (1.42-2.59)
Ireland	63 (57-68)	2.74 (2.51-2.97)	108 (93-124)	2.67 (2.3-3.04)
Israel	60 (55-67)	2.28 (2.06-2.5)	180 (156-202)	2.72 (2.39-3.07)
Italy	672 (639-694)	1.29 (1.23-1.33)	1822 (1593-1958)	2.24 (2.03-2.39)
Jamaica	29 (26-32)	3 (2.65-3.34)	110 (87-135)	7.04 (5.58-8.76)
Japan	1701 (1594-1759)	1.78 (1.67-1.84)	2970 (2465-3256)	1.7 (1.52-1.82)
Jordan	17 (13-24)	2.77 (2.07-3.93)	58 (44-75)	2.07 (1.57-2.69)
Kazakhstan	410 (381-443)	5.25 (4.88-5.65)	336 (287-387)	3.27 (2.8-3.76)
Kenya	46 (34-65)	1.14 (0.85-1.61)	157 (116-225)	1.45 (1.08-2.03)
Kiribati	1 (1-1)	5.2 (2.45-6.9)	2 (1-3)	5.06 (2.18-6.85)
Kuwait	4 (4-5)	2.01 (1.69-2.38)	20 (16-25)	2.21 (1.74-2.75)
Kyrgyzstan	55 (49-61)	3.02 (2.7-3.37)	73 (61-86)	2.79 (2.34-3.28)
Lao People's Democratic Republic	43 (25-64)	3.8 (2.28-5.56)	71 (44-97)	3.02 (2-4.07)
Latvia	88 (81-96)	3.77 (3.48-4.08)	115 (89-147)	4.46 (3.41-5.76)
Lebanon	34 (26-44)	3.1 (2.43-4.06)	73 (52-101)	2.56 (1.82-3.56)
Lesotho	11 (9-15)	2.07 (1.58-2.74)	28 (17-41)	3.91 (2.49-5.65)

Liberia	12 (9-17)	2.48 (1.87-3.33)	22 (14-31)	2.39 (1.61-3.4)
Libya	16 (11-21)	1.86 (1.24-2.49)	45 (30-61)	1.88 (1.22-2.54)
Lithuania	118 (109-127)	4.23 (3.93-4.54)	150 (121-184)	4.13 (3.31-5.1)
Luxembourg	16 (15-18)	4.91 (4.47-5.35)	17 (14-20)	3.01 (2.51-3.5)
Madagascar	59 (36-73)	2.38 (1.43-2.96)	128 (77-181)	2.41 (1.41-3.38)
Malawi	32 (24-41)	1.63 (1.21-2.07)	54 (39-72)	1.43 (1.04-1.88)
Malaysia	119 (87-148)	2.61 (1.9-3.24)	339 (231-446)	2.61 (1.78-3.42)
Maldives	1 (0-1)	2.2 (1.13-3.16)	2 (1-2)	1.17 (0.91-1.5)
Mali	37 (29-49)	1.81 (1.4-2.38)	68 (51-95)	1.72 (1.32-2.37)
Malta	8 (8-9)	3.53 (3.15-3.95)	14 (11-16)	2.61 (2.14-3.1)
Marshall Islands	0 (0-1)	5.2 (3.33-6.83)	1 (1-2)	6.66 (3.55-9.65)
Mauritania	16 (12-22)	3.25 (2.33-4.34)	26 (18-35)	2.67 (1.86-3.57)
Mauritius	19 (17-21)	4.91 (4.47-5.4)	33 (27-40)	3.47 (2.83-4.24)
Mexico	343 (330-353)	1.62 (1.55-1.67)	923 (760-1121)	1.47 (1.22-1.79)
Micronesia (Federated States of)	1 (1-2)	6.08 (3.73-8.42)	3 (1-4)	7.02 (3.61-10.38)
Monaco	1 (0-1)	1.23 (0.87-1.69)	1 (1-1)	1.28 (0.9-1.64)
Mongolia	16 (12-21)	2.51 (1.88-3.44)	31 (22-42)	2.2 (1.57-3)
Montenegro	10 (8-13)	2.83 (2.33-3.87)	16 (13-22)	2.98 (2.4-4.08)
Morocco	100 (76-128)	1.48 (1.12-1.88)	265 (183-348)	1.71 (1.19-2.23)
Mozambique	76 (45-107)	2.53 (1.51-3.55)	160 (96-235)	2.81 (1.68-4.09)
Myanmar	412 (251-616)	3.21 (2.05-4.71)	697 (490-913)	2.62 (1.92-3.38)
Namibia	8 (6-12)	2.16 (1.63-3.03)	18 (12-25)	2.3 (1.6-3.22)
Nauru	0 (0-0)	6.74 (3.88-9.48)	0 (0-0)	6.88 (3.26-9.6)
Nepal	60 (37-109)	1.34 (0.81-2.44)	150 (96-329)	1.34 (0.86-2.97)
Netherlands	335 (309-358)	2.79 (2.59-2.96)	593 (518-667)	3.03 (2.67-3.39)
New Zealand	67 (61-72)	3.05 (2.81-3.3)	130 (116-145)	3.07 (2.74-3.41)

Nicaragua	9 (7-12)	1.09 (0.9-1.47)	39 (30-50)	1.65 (1.27-2.16)
Niger	26 (18-37)	2.12 (1.44-3)	78 (57-108)	2.12 (1.54-2.87)
Nigeria	143 (85-331)	0.7 (0.42-1.64)	333 (206-718)	0.8 (0.51-1.71)
Niue	0 (0-0)	4.54 (2.66-6.16)	0 (0-0)	4.6 (2.27-6.38)
North Macedonia	40 (33-63)	4.07 (3.39-6.63)	86 (64-111)	5.14 (3.87-6.65)
Northern Mariana Islands	0 (0-1)	5.58 (3.81-10.77)	2 (1-3)	7.66 (5.1-9.7)
Norway	125 (116-131)	3.14 (2.94-3.27)	149 (132-163)	2.73 (2.47-3)
Oman	3 (2-4)	0.93 (0.64-1.32)	6 (4-8)	0.95 (0.69-1.19)
Pakistan	865 (688-1179)	3.48 (2.73-4.84)	2491 (1826-3412)	4.86 (3.64-6.6)
Palau	0 (0-0)	0.55 (0.39-0.76)	0 (0-0)	0.52 (0.38-0.72)
Palestine	17 (11-24)	3.56 (2.46-4.97)	47 (28-59)	4.07 (2.54-5.09)
Panama	13 (11-14)	1.77 (1.57-1.98)	57 (44-74)	2.68 (2.07-3.48)
Papua New Guinea	27 (17-37)	2.94 (1.94-4.01)	84 (46-122)	3.66 (2.06-5.23)
Paraguay	48 (33-57)	4.15 (2.91-4.97)	92 (66-148)	3.19 (2.29-5.18)
Peru	226 (183-276)	3.73 (3.04-4.6)	473 (343-643)	2.84 (2.05-3.87)
Philippines	453 (347-531)	2.87 (2.37-3.58)	1114 (822-1498)	2.58 (1.92-3.57)
Poland	1064 (1020-1101)	4.12 (3.95-4.26)	1775 (1415-2207)	4.19 (3.31-5.24)
Portugal	292 (268-317)	3.67 (3.38-3.95)	379 (331-432)	2.69 (2.38-3.02)
Puerto Rico	51 (46-56)	2.61 (2.36-2.86)	104 (81-132)	2.67 (2.08-3.41)
Qatar	1 (0-1)	1.84 (0.89-2.66)	3 (1-5)	2.51 (1.01-3.47)
Republic of Korea	475 (231-548)	2.68 (1.35-3.08)	373 (289-463)	0.77 (0.59-0.94)
Republic of Moldova	91 (83-98)	3.41 (3.15-3.66)	101 (86-118)	2.95 (2.51-3.45)
Romania	523 (492-558)	3.36 (3.16-3.59)	594 (483-720)	2.89 (2.34-3.52)
Russian Federation	5532 (5324-5759)	4.71 (4.52-4.91)	5994 (4975-7075)	4.06 (3.36-4.82)
Rwanda	52 (31-69)	3.29 (2.02-4.31)	82 (55-107)	2.53 (1.65-3.26)
Saint Kitts and Nevis	2 (1-2)	7.53 (6.71-8.43)	2 (2-3)	6.63 (5.44-8.03)

Saint Lucia	2 (2-2)	4.59 (4.16-5.04)	5 (4-6)	4.56 (3.81-5.42)
Saint Vincent and the Grenadines	3 (2-3)	6.45 (5.81-7.05)	5 (5-6)	8.08 (7.02-9.29)
Samoa	2 (1-3)	4.5 (3.29-6.13)	3 (2-4)	4.59 (3.21-6.15)
San Marino	0 (0-0)	0.83 (0.65-1.04)	0 (0-0)	0.83 (0.52-1.31)
Sao Tome and Principe	1 (1-2)	3.82 (2.86-4.81)	2 (1-3)	4.79 (2.95-6.56)
Saudi Arabia	28 (19-43)	1.22 (0.82-1.81)	105 (71-141)	1.59 (0.98-2.12)
Senegal	34 (26-47)	2.28 (1.75-3.08)	90 (66-120)	2.47 (1.84-3.24)
Serbia	227 (184-314)	3.8 (3.09-5.28)	368 (256-486)	4.19 (2.95-5.53)
Seychelles	1 (1-1)	2.95 (2.41-3.67)	2 (1-2)	3.05 (2.41-3.8)
Sierra Leone	17 (13-24)	1.95 (1.46-2.65)	40 (27-55)	2.37 (1.62-3.29)
Singapore	16 (15-18)	1.41 (1.26-1.56)	78 (68-88)	1.92 (1.66-2.17)
Slovakia	200 (161-231)	5.76 (4.65-6.62)	245 (168-324)	4.51 (3.11-5.94)
Slovenia	60 (47-77)	4.02 (3.14-5.13)	79 (60-102)	3 (2.24-3.9)
Solomon Islands	4 (2-6)	5.9 (3.83-8.28)	12 (7-18)	7.53 (4.16-10.46)
Somalia	34 (20-52)	2.64 (1.53-3.94)	91 (49-148)	2.54 (1.38-4.09)
South Africa	207 (162-252)	1.78 (1.38-2.17)	569 (395-675)	2.3 (1.58-2.72)
South Sudan	28 (16-41)	2.75 (1.6-4.07)	41 (25-61)	2.46 (1.52-3.65)
Spain	897 (821-957)	2.85 (2.62-3.03)	1609 (1395-1798)	2.81 (2.47-3.13)
Sri Lanka	77 (62-114)	1.54 (1.25-2.22)	244 (162-334)	1.76 (1.16-2.41)
Sudan	39 (25-69)	0.88 (0.58-1.6)	84 (55-138)	0.98 (0.66-1.62)
Suriname	3 (2-4)	2.15 (1.78-2.87)	9 (6-11)	2.65 (1.96-3.32)
Sweden	260 (237-281)	2.91 (2.67-3.12)	316 (276-353)	2.53 (2.24-2.8)
Switzerland	159 (145-173)	2.5 (2.29-2.71)	189 (161-215)	1.84 (1.6-2.08)
Syrian Arab Republic	31 (22-47)	1.22 (0.87-1.99)	62 (43-105)	1.11 (0.78-1.93)
Taiwan (Province of China)	67 (62-73)	0.96 (0.88-1.03)	343 (268-444)	1.64 (1.27-2.14)
Tajikistan	42 (35-56)	2.68 (2.25-3.6)	110 (73-143)	3.91 (2.69-5.02)

Thailand	285 (209-344)	1.48 (1.11-1.79)	634 (431-859)	1.13 (0.77-1.53)
Timor-Leste	4 (3-6)	2.91 (1.82-4.07)	12 (7-16)	2.83 (1.85-3.71)
Togo	14 (11-18)	2.33 (1.8-3)	43 (30-60)	2.26 (1.59-3.09)
Tokelau	0 (0-0)	5.55 (3.34-7.62)	0 (0-0)	5.55 (2.73-7.83)
Tonga	1 (1-1)	3.35 (2.04-4.9)	2 (1-2)	3.6 (1.82-5.57)
Trinidad and Tobago	23 (22-26)	5.42 (4.96-5.92)	63 (48-81)	6.38 (4.84-8.27)
Tunisia	28 (21-35)	1.21 (0.9-1.52)	72 (49-100)	1.13 (0.78-1.58)
Turkey	523 (338-689)	2.83 (1.83-3.72)	1029 (625-1321)	2.22 (1.36-2.85)
Turkmenistan	29 (26-31)	2.56 (2.34-2.8)	20 (16-26)	0.91 (0.71-1.16)
Tuvalu	0 (0-0)	5.56 (3.42-7.62)	0 (0-0)	5.47 (2.82-7.96)
Uganda	83 (59-121)	2.64 (1.9-3.88)	299 (220-386)	4.08 (3.04-5.26)
Ukraine	1916 (1728-2104)	4.13 (3.76-4.54)	1803 (1405-2243)	3.89 (3.02-4.86)
United Arab Emirates	3 (1-5)	1.93 (0.71-3.96)	16 (7-27)	1.5 (0.58-2.8)
United Kingdom	1393 (1312-1435)	2.58 (2.45-2.65)	2302 (2092-2433)	3.23 (2.98-3.4)
United Republic of Tanzania	145 (87-189)	2.73 (1.62-3.54)	342 (218-435)	2.83 (1.8-3.58)
United States of America	5614 (5269-5814)	2.93 (2.77-3.02)	10260 (9538-10776)	3.3 (3.11-3.45)
United States Virgin Islands	2 (2-2)	4.24 (3.39-5.46)	4 (3-5)	4.04 (3.11-5.07)
Uruguay	69 (63-75)	3.06 (2.8-3.34)	81 (71-92)	2.43 (2.14-2.77)
Uzbekistan	151 (138-164)	2.29 (2.11-2.49)	343 (281-416)	2.62 (2.19-3.13)
Vanuatu	1 (1-2)	4.04 (2.26-6.03)	4 (2-6)	5.07 (2.53-7.66)
Venezuela (Bolivarian Republic of)	184 (170-198)	3.71 (3.42-3.98)	426 (324-560)	2.75 (2.09-3.61)
Viet Nam	285 (214-360)	1.24 (0.93-1.57)	677 (451-901)	1.31 (0.88-1.72)
Yemen	22 (13-42)	0.87 (0.53-1.72)	66 (46-110)	0.98 (0.69-1.67)
Zambia	46 (28-59)	3.46 (2.13-4.44)	102 (63-141)	3.09 (1.91-4.26)
Zimbabwe	73 (56-92)	3.69 (2.84-4.58)	214 (125-297)	5.49 (3.33-7.51)

ASDR: age-standardized death rate
